# Things are looking (farther) up: Upward gaze orientation is overestimated

**DOI:** 10.3758/s13414-026-03268-x

**Published:** 2026-04-29

**Authors:** Dennis M. Shaffer, Montse Juarez, Alexis Shaver, Carissa Brown, Brooke Hill, Ryanne E. Shaffer

**Affiliations:** Department of Psychology, The Ohio State University Mansfield, 1760 University Drive, Mansfield, OH 44906 USA

**Keywords:** 3D perception, Space Perception, Spatial Vision, Visual perception

## Abstract

In the current work, we examine upward head orientation. Previous work has shown that downward gaze orientation is overestimated by a factor of ~1.5, while azimuth gaze orientation is overestimated by a factor of ~1.2. In Experiment 1, we compared head orientation in the upward, downward, and azimuth (leftward and rightward) directions. The factor by which observers overestimated upward head orientation was 1.56, similar to overestimates in downward head orientation in this and downward gaze orientation in previous work. Azimuth gaze orientation was overestimated by ~1.26, similar to previous work. In Experiment 2, we examined upward head orientation with a smaller range of angles and found that the factor increased slightly (1.7) but not statistically so from Experiment 1 or from previous work. We also examined the perceptual head orientation boundaries for upward and downward gaze orientation. These indicate a far more limited range than 90° up and down. In Experiment 3, participants gave head orientation estimates while blindfolded. Their estimates of upward, downward, and azimuth head orientation matched our current and others’ previous work. Independent of gaze, head orientation is overestimated by the same factor as is gaze orientation. The upward head orientation overestimates predict well observers’ estimates of heights of familiar suspended objects and fits with why some familiar suspended objects appear smaller than actual. The common scale expansion for upward and downward head and gaze orientation is a useful perceptual regularity that reliably predicts how we spatially map the environment and how we perceive the world.

## Introduction

It is well established that observers overestimate visually, haptically, pedally, and proprioceptively perceived geographical, virtual, and man-made hills by between 5° and 25° (Bridgeman & Cooke, 2015; Durgin & Li, 2011; Hajnal et al., 2011; Li & Durgin, 2009, 2010; Proffitt et al., 1995; Shaffer & Flint, 2011; Shaffer et al., 2019; Shaffer & McManama, 2015; Shaffer et al., 2016a). Observers’ estimates of slant seem to be best explained by a combination of perceived gaze orientation combined with perceived optical slant – an estimate of surface orientation with respect to the line of gaze (Bridgeman & Cooke, 2015; Chiu et al., 2014; Li & Durgin, 2009, 2010; Ross, 1994).

In the last 10–15 years or so it has been shown that perception of gaze orientation is also not veridical (Durgin & Li, 2011; Klein et al., 2016; Li & Durgin, 2009, 2016). For instance, Durgin and Li (2011, Experiment 1) had participants stand in an open field and verbally report gaze direction while looking at targets arranged from 4° to 45° along a sloped surface. Slope estimates fit a linear function, with a gain of 1.53 indicating that observers were overestimating their head orientation. So when orienting their head at 30° they perceived they were orienting their head at ~45° (or perceived it to be 1.5 times more tilted than actual). In a nonverbal task requiring bisection of gaze angle between horizontal and vertical, the overall mean angle participants estimated was 31°, suggesting that when their head was tilted downward at 31° they felt like it was tilted at 45°, again supporting the idea that gaze declination is overestimated by a factor of ~1.5.

Li and Durgin (2009) found that misperception of head orientation (and thus gaze orientation) could help explain the misperception of downhill surface orientation. In their Experiment 3A participants matched a line to planar wood surfaces presented at three different orientations. A model with an overall gain of 1.5 best fit the data. In their Experiment 3B they had participants give verbal estimates to various downward-sloped (from their perspective) landmarks ranging in orientation from 8.5° to 43.3°. Estimates were modeled by a linear fit with a gain of 1.5. This work supports the idea that gaze declination is overestimated by a factor of ~1.5.

Misperception of gaze orientation also has interesting implications for perception of slanted surfaces and ground extents in the depth direction. For instance, the overestimation of gaze orientation found in Durgin and Li (2011) and Li and Durgin (2009) matched what they and others have modeled and found with the overestimation of upward slant by a factor of ~1.5 for outdoor hills, virtual hills, haptic estimations of slant, and body orientation (Durgin & Li, 2011, Li & Durgin, 2010; Shaffer & Flint, 2011; Shaffer et al., 2016a, 2019). So why might these two be connected? If one overestimates one’s downward gaze orientation by a factor of 1.5, then a flat ground surface should look as if it is tilted downward by the same factor unless we correct for it by misperceiving (*over*estimating) optical (or geographical) slant of surfaces by the same factor (1.5), which previous work shows that we do (especially for angles less than 60°) (Durgin & Li, 2011; Durgin et al., 2010; Li & Durgin, 2010; Shaffer & Flint, 2011). These two biases leave the ground surface appearing flat (Li & Durgin, 2010).

Regarding distance perception, the underestimation of distance in the depth direction compared to that in the sagittal direction may also be explained by the combined bias of misperceived gaze declination accompanied by misperceived optical slant (Durgin & Li, 2011). Durgin and Li (2011) and Keezing and Durgin (2018) showed that this combined bias leaves people looking at a given distance as closer to their feet due to overestimation of gaze declination. The overestimation of gaze declination, in fact, predicts an underestimation of ground distance by about 0.7. This underestimation of ground distance extents fits extant data very well (see Durgin & Li, 2011, for their comparison of their angular declination work to the measured distance aspect ratios between depth and sagittal extents of Loomis et al., 1992, and Loomis & Philbeck, 1999, which fit rather closely to a perceived angular declination gaze of 1.5 (1.6 and 1.56, respectively)). Consistent with this, Li et al. (2011, Experiment 1) showed that when asked to place themselves at a distance in depth away from a frontal distance between two experimenters, participants placed themselves too far away, consistent with a perceived egocentric distance compression in the depth direction. The gain that they found (ratio of depth distance to frontal distance) was 1.43, consistent with this perceived scale expansion of space due to perceived gaze declination.

While there has been much work revealing overestimation of downward gaze direction (relative to gaze straight ahead), which explains overestimates of slant and underestimation of distance in the depth direction compared to frontal distances, work has also focused on gaze perception in the upward direction, relative to gaze straight ahead (Durgin & Li, 2011; Klein et al., 2016; Tardeh et al., 2024). For instance, Durgin and Li (2011) found (in their Experiment 4) that when they elevated and lowered gaze directions, gaze lowered estimates were 1.46, similar to the gain of the verbal estimates across the eight sloped surfaces they examined (gain = 1.42, excluding the 22.5° gaze elevated condition). Their Fig. 7b (p. 1865) suggests that gaze inclination in the elevation coordinate may be overestimated by a similar factor to that of gaze declination, though this was never directly experimentally tested. However, their data suggest that gaze inclination may be overestimated and also be overestimated by an approximately equivalent magnitude by which gaze declination is overestimated. For instance, Li et al. (2011, Experiment 2) had participants perform a pole-matching task to four poles that varied in height from 3.75 to 22.5 m. Participants then adjusted their forward/backward distance to the pole until it matched the height of the target. The matching estimates fit well to a linear model with a gain of 1.5 (depth distance: vertical distance). Klein et al. (2016) performed a similar task with a 10-m lamp post (Experiment 1) and found that a model with a gain of 1.47 fit estimates of overestimation in the vertical direction well. Klein et al. (2016, also Experiment 1) also performed an angular direction task asking their participants to adjust their position until they felt like they were looking (their gaze was oriented) at 45° when looking at a 35-m tall column holding a water tank. Participants stopped when their gaze was oriented at 30.7°, suggesting a gaze inclination expansion by a factor of ~1.5. Tardeh et al. (2024) investigated whether angular expansion/perceived elevation was dependent on whether a ground plane was present. Depending on whether a ground plane was present, where it was positioned on the screen, and whether participants were seated or supine, gains when looking upward varied from 1.2 to 1.68. They mention that the data were difficult to interpret in Experiment 3, where the gain was 1.2 as the variability was so large and one of the targets was located at 60°. Similar work has found a similar depth distance on the ground: height gains of 1.67 (Higashiyama & Ueyama, 1988) and 1.47 and 1.78 (Kammann, 1967, Experiment 1A and Experiment 1B, respectively). Yet other work using matching tasks has found overestimates of heights by up to 30% depending on the distance of the observer to the target (Stefanucci & Proffitt, 2009).

While much work has explored upward and downward gaze orientation (e.g., Durgin & Li, 2011; Klein et al., 2016; Li & Durgin, 2009; Li et al., 2011; Tardeh et al., 2024) and less work has been performed measuring azimuth gaze orientation, Li and Durgin (2016) showed that azimuth gaze overestimates have a gain of 1.26, but mention they did not directly compare this with perceived gaze inclination. We wanted to further explore this work in five novel ways. First, we wanted to evaluate gaze as measured by head-pitch orientation with instruction to keep gaze forward relative to the head. Previous work has shown overestimations of a factor of ~1.5 for estimated pitch of the body, foot, and hand (Hajnal et al., 2011; Shaffer et al., 2016a, 2016b, 2019). We wanted to evaluate the contribution of head-pitch orientation with gaze straight ahead as a proxy for gaze orientation. Second, we wanted to extend the evaluation of gaze orientation as measured by head-pitch orientation to the estimates of orientation in the azimuth and elevation (up and down) coordinates within the same observers. This may give us somewhat more precise and reliable indicators of how people mis(perceive) directly measured gaze orientation in all three directions as the same participants are giving estimates in each of three directions. Third, we wanted to evaluate head-pitch orientation independent of vision. Does orienting one’s head independent of vision exaggerate overestimations of gaze similar to the findings by Hajnal et al. (2011) comparing verbal to proprioceptive estimates when participants stood on a slope (where proprioceptive judgments were elevated compared to verbal judgments), or are verbal estimates of head-pitch orientation similar whether eyes are open or closed, similar to the findings of Shaffer at al. (2016b), where estimates of how much participants were tilted backward were similar with eyes open to eyes closed? Fourth, we wanted to test whether these overestimates might predict and explain overestimates of vertical extents when matched to horizontal extents (cf. Klein et al., 2016). Finally, we wanted to define the perceptual boundaries of head orientation in the elevation coordinate plane (upward and downward).

More specifically, the goals of the current work were to:

1. Investigate estimates of head pitch in the upward direction in two ways: First, by having participants verbally estimate at what orientation their head was positioned when it is moved to one of three different orientations, and second, by having participants move their head to three different verbally given orientations in the elevation coordinate higher than looking straight ahead.
2. Investigate estimates of head pitch in the azimuth and downward directions within the same observers to directly compare these estimates to head pitch in the upward direction.
3. Define the perceptual boundaries of functional head pitch in the elevation coordinate plane (this includes upward and downward head pitch).
4. Compare estimates of head pitch to depth distances on the ground: vertical matches and height estimates found in extant work.
5. Evaluate the independent contribution of head pitch (with and without vision).
6. Compare estimates of head pitch to verbal estimates of the height of an object from memory.

**Fig. 1:**
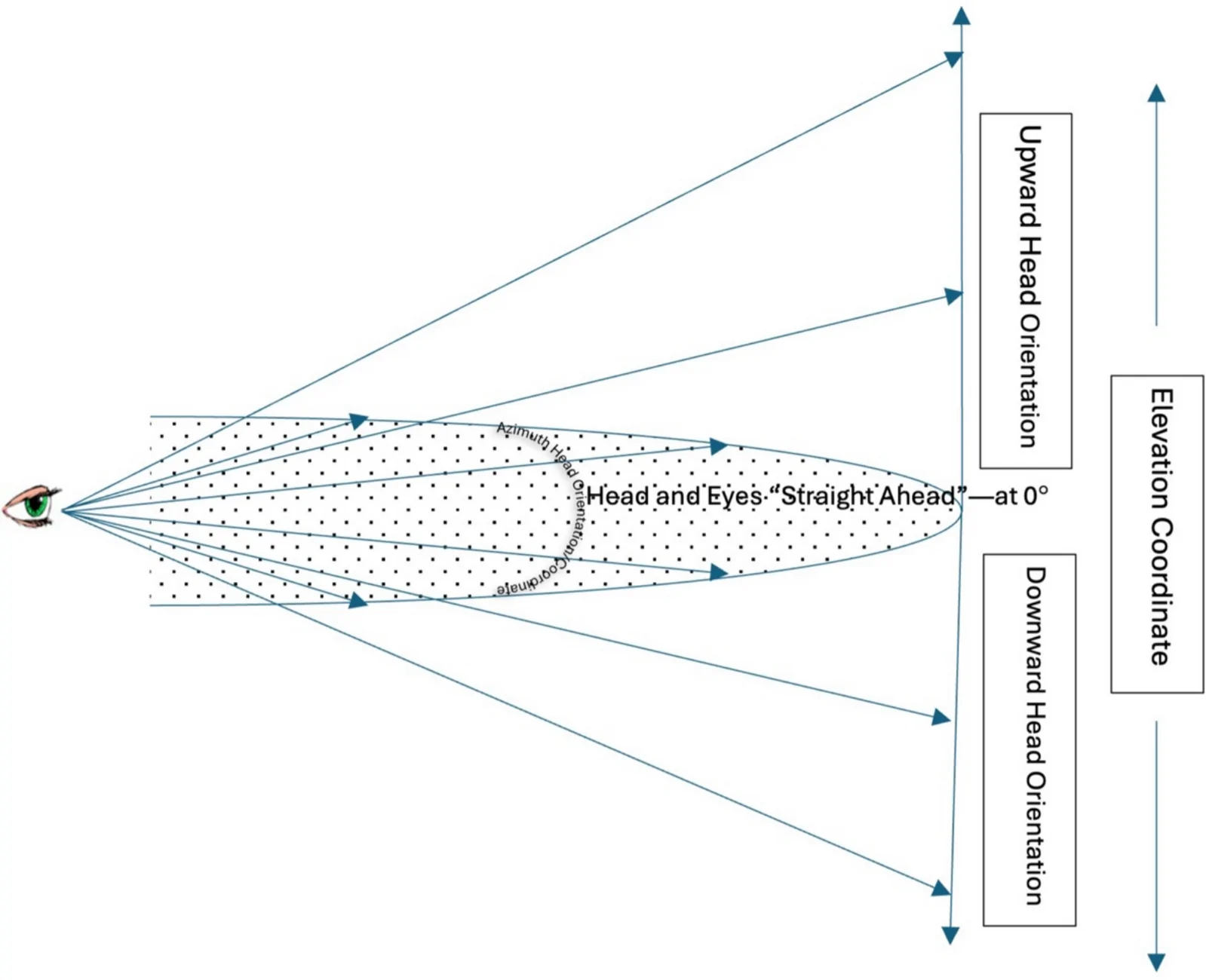
Here we define directions of head orientation for the current paper. “Elevation” may refer to any up or down gaze, while we define “**upward**” and“**downward**” from a set position of straight ahead – eyes and head are perfectly parallel to flat ground while head and body are facing straight ahead. Azimuth head orientation is head oriented to the left or right of facing straight ahead

Figure 1 shows what we are referring to when we say “upward,” “downward,” and azimuth. These are dependent on a sitting or standing observer looking straight ahead. We are more interested in clarifying that to which we are referring when we use these terms in the current paper than defining these terms generally. For instance, when we refer to “upward,” we are referring to head oriented up from looking straight ahead or from 0°, and not from any position lower than that. Similarly, when we refer to “downward,” we are referring to head oriented down from looking straight ahead or from 0°, and not from any position higher than that.

## Experiments 1a, 1b, 1c, and 1d

### Method

#### Participants

Thirty participants (18 female) whose mean age was 18.6 years (*SD* = 0.89) from the Ohio State University at Mansfield participated in fulfillment of an Introductory Psychology requirement. Each participant was required to sign a consent form for the study. Informed consent was obtained for all participants. This study was approved by The Ohio State University Behavioral and Social Sciences Institutional Review Board (Study Number: 2023B0282).

#### Power analyses

Two factors informed our decision as to the number of participants to use in Experiments 1 and 2 (Lakens, 2022). First, 30 participants were used in Experiments 1a–d and 33 in Experiments 2a and b based on previous studies performed that most closely resemble the approach and methods of these studies – having participants estimate their head/gaze orientations – and also statistically used, at least in part, a repeated-measures design. The number of participants used in each of these studies was 35 (Li & Durgin, 2016, Experiment 1), 19 (Li & Durgin, 2016, Experiment 2), 13 (Li & Durgin, 2009, Experiment 2B), and eight (Li & Durgin, 2009, Experiment 3B). In all of these experiments statistically significant effects were found.

Second, in our analyses, we first planned to perform a one-way repeated-measures ANOVA.^1^ In order to determine the proper number of participants, we used the results of Li and Durgin (2016, Experiment 1) as a guide for the effect size to use for our preliminary analysis. In that work they used three different gaze conditions, and found *F* = 7.9 with *n* = 35. We converted these results to Cohen’s* f* =.4395.^2^ Using an effect size of *f* =.4395, *α* =.05, Power =.95, one group with four measurements, correlation among repeated measures = 0.50, and a nonsphericity correction ε = 1, the total sample size necessary would be *n* = 13. For this power analysis we used G*Power 3.1.9.6 (Faul et al., 2007) and for the design we used the “*F*-tests: ANOVA: Repeated measures, within factors” procedure and the Type of power analysis was “A priori: Compute required sample size – given α power, and effect size.”

For each condition, we also planned to perform a one-sample *t*-test for comparing an index of participants’ overestimations to a value of 1, indicating veridical perception for each condition. The number of participants in two experiments that closely match not only the methodology and focus of this study – having people verbally estimate different orientation of their hand or orient them to a verbally given angle (Shaffer et al, 2016a, Experiments 1 and 3), but also the analysis – using one-sample *t*-tests to analyze estimates compared to actual orientations, was 30 in each experiment. In both of these experiments statistically significant effects were found. In order to determine the proper number of participants to use, we used the results of Shaffer et al. (2016a) as a guide for the effect size to use for our preliminary analysis. In those experiments, effect sizes of Cohen’s* d* =.18,.57,.97, 1.39, 1.22 were found. We took the mean Cohen’s* d* across those experiments and found an average effect size of Cohen’s* d* =.866. Using a one-tailed test (participants will overestimate (Experiments 1a, c, and d) or underestimate (Experiment 1b) the actual head orientation, an effect size of *d* =.866, *α* =.05, Power =.95, the total sample size necessary would be *n* = 16. For this power analysis we used G*Power 3.1.9.6 (Faul et al., 2007) and for the design we used the “*t*-tests: Means: Difference from constant (one-sample case)” procedure and the Type of power analysis was “A priori: Compute required sample size – given α power, and effect size.”

#### Sensitivity power analyses

Consistent with the recommendations of Giner-Sorolla et al. (2024) and Lakens (2022), once we decided on a sample size of 30 due to previous studies and the aforementioned a priori analysis, we performed an effect size sensitivity analysis in order to determine the minimal effect it would take to detect a difference among the four conditions. Using α =.05, Power =.95, *n* = 30, one group with four measurements, correlation among repeated measures = 0.50, and a nonsphericity correction = 1, the minimal effect our study would detect is *f* =.2736 (effect sizes of *f* =.218 and.249 can be detected for Power = 80% and 90%, respectively (Lakens, 2022)).^3^ For this power analysis we used G*Power 3.1.9.6 (Faul et al., 2007) and for the design we used the “*F*-tests: ANOVA, repeated-measures, within-factors” procedure. The following sections describe the analyses performed condition by condition.

We performed a second effect size sensitivity analysis in order to determine the minimal effect it would take for this analysis to detect a difference between an index of participants’ overestimates (Experiments 1a, c, and d) or underestimates (Experiment 1b) and a value of 1 (veridical perception). This analysis had sensitivity power using a one-tailed test (participants would verbally overestimate/underestimate orientations), ε =.05, Power =.95, and *n* = 30, the minimal effect our study would detect is *d* =.62 (effect sizes of *d* =.465 and.547 can be detected for Power = 80% and 90%, respectively (Lakens, 2022)). For this power analysis we used G*Power 3.1.9.6 (Faul et al., 2007) and for the design we used the “t-tests: Means: Difference from constant (one-sample case)” procedure.

#### Design

Experiments 1a–d were composed of four within-subjects conditions. In Experiment 1a, researchers oriented the participant’s head at three different head orientations (18°, 40°, and 62°) in the upward direction, and participants verbally estimated their head orientation. In previous work, it has been shown that participants are able to report perceived gaze and head orientation (Durgin & Li, 2011; Li & Durgin, 2009). In Experiment 1b, participants were verbally given three angles at which to orient their head in the upward direction (22°, 45°, and 67°). In Experiment 1c, researchers oriented the participant’s head at three different orientations (18°, 40°, and 62°) in the downward direction, and participants verbally estimated their head orientation, and in Experiment 1d, researchers oriented the participant’s head at three different orientations (18°, 40°, and 62°) in the azimuth (left or right) direction. We randomly assigned participants to either head to the left or head to the right for all three head orientations.

#### Materials

A digital magnetic angle locator was secured on a bicycle helmet that participants wore to measure head orientation for Experiments 1a–c. A magnetic angle inclinometer was secured on a bicycle helmet that participants wore to measure head orientation for Experiment 1d. In both upward conditions and the azimuth condition people were looking at a flat white background of walls (forward and left and right). When looking in front, many participants looked upward enough to be looking at an all-white ceiling. When looking downward, many participants looked downward far enough to be looking at an all light blue carpet on the floor.

#### Procedure

All participants took part in every condition. This was done to more directly compare upward, azimuth, and downward head-orientation perception. The order of the four conditions was randomized for each participant as were the presentation order of the three angles used within each condition. Participants were first seated in a laboratory in a chair that allowed them to freely move their head, neck, and shoulders. We secured a bicycle helmet to their head. We next had them look straight ahead and orient their head at 0°. In three conditions (Experiments 1a, 1c, and 1d) we oriented their head and they verbally estimated their head orientation (with eyes straight ahead). In one of the two upward head orientation conditions (Experiment 1b), participants were verbally given an orientation at which to orient their head and they did so. After the participant moved their head to the requested orientation or the researcher moved their head to a given orientation and the participant gave their estimate, participants closed their eyes prior to moving their head to the subsequent orientation. They did this until they estimated all three orientations in that condition. We had them estimate a subsequent orientation from the position of the previous orientation instead of having them start from the same orientation to avoid anchoring biases (Shaffer et al., 2014, 2015). We also told participants to look straight ahead with their eyes as their head moved. We stationed a researcher in front of them to make sure they did this. Therefore, what we were measuring was head-pitch orientation with eyes looking straight ahead. Participants were told that if their head was level and they were looking straight ahead that would be considered an orientation of 0° and if the head was oriented so they were looking directly above them on the ceiling in line with their torso that would be considered an orientation of 90°. We did not proceed until all participants clearly understood this.

### Results

Prior to analyzing the data, we removed any verbal estimates (for Experiments 1a, 1c, and 1d) where the angle of 18° was estimated at or greater than 60°. This is because much of the downward gaze-orientation research has shown gains of 1.5 and some of the work on perceiving slanted surfaces shows that climbers cannot distinguish between hills of ~60° to 90°. This means that someone who gave a verbal estimate of greater than 60° in any direction when their head was at 18° perceived their head as tilted maximally. Also given that all participants were instructed as to what 0° and 90° is and the restrictions of the head and neck (especially in upward and downward directions), it would seem that estimates of 18° that equaled or exceeded 60° are not as much overestimates of head orientation as they are a misunderstanding of what participants were asked to do. For these cases, we simply removed that estimate but kept the remaining estimates for the 40° and 62° angles for that participant.

#### Converting estimates to gains for all tasks

In previous work (e.g., Shaffer et al., 2016a, 2016b, 2019), in order to test the pattern of people’s estimates across different angles, we plotted the estimates for each of the different angles for each participant against the orientations they were asked to estimate. We then calculated and recorded the slope of the best fitting line for each participant. In the present work, many people estimated 18° as between 30° and 35°. Due to this, the maximum slope a line could have, even if the average estimate for 62° being 90° is between 1.25 and 1.35. It is for this reason that we instead calculated a gain (a ratio of head orientation estimate/actual head orientation) for each angle estimated for every participant. We then averaged these gains across angles for each participant and performed analyses on those averaged gains (one gain for each participant).

For the condition where participants oriented their head to a verbally given angle, an overestimate of head orientation by a factor of 1.5 in upward head orientation would mean that if someone were asked to estimate their head orientation when we positioned their head at 30°, they would estimate it to be 45° (or their verbal estimate would be 1.5 times as great as the head orientation we asked them to estimate). Drawing from previous experiments, the mean gain for upward head orientation is ~1.5, which means that if we oriented their head at 30°, they would estimate it to be ~45°. This also means that for what we asked of participants in the current experiment – to orient their head at a verbally given angle – we should expect the inverse result. That is, if they overestimate upward head orientation by a factor of 1.5 consistent with previous work, when we ask them to orient their head at 30°, 30° should feel like 45°, and their gain of where they orient their head should be 30°/45° =.67, much smaller than a value of 1.

#### Comparing Gains Across Head Orientation Tasks

We performed Bayesian analyses throughout the Results sections for all experiments as there were several times we hypothesized null results and wanted to be able to show evidence in favor of no differences. Therefore, we used Bayesian analyses for all analyses, whether or not we were testing for differences or no differences to be consistent throughout the paper consistent with the recommendations of both Dienes (2924) and Kruschke (2021).

We converted observers’ verbal estimates of where their head was oriented versus where it was actually oriented into a gain across the three angles they were asked to estimate (verbal estimate of head orientation/actual head orientation). Prior to analyzing each task, we first performed a Bayesian repeated-measures ANOVA comparing gains across the four different tasks using *JASP *(Version 0.19.2). The ANOVA showed that average gains were significantly different across tasks, *BF*_*10*_ = 3.186 × 10^24^ (decisive evidence for an effect), *F*(3, 87) = 68.46, *p* <.001, η^2^ = 0.702, Noninformative prior =.5. Post hoc tests indicated that gains for upward and downward orienting of the head were statistically equivalent (at ~1.56–1.69.56.69), *BF*_*10*_ = 2.275, while the average gains for reproduced verbally given angles (~0.6) and azimuth head orientation estimates (~1.25) were significantly less than upward and downward gains), *BF*_*10Upward-VerballyGiven*_ = 3.14 × 10^8^ (decisive evidence), *BF*_*10Downward-VerballyGiven*_ = 4.76 × 10^12^ (decisive evidence), *BF*_*10Upward-Azimuth*_ = 22.17 (strong evidence), and *BF*_*10Downward-Azimuth*_ = 19924.12 (decisive evidence), Noninformative prior =.414.^4^ Credible intervals for each condition are given in each of the subsequent analyses.

#### Experiment 1a: Verbal estimates of upward head orientations (18°, 40°, 62°)

Two estimates of 18° equaled or exceeded 60° (60°, 75°), so these two estimates of 18° were removed from the analyses. For the two participants whose 18° estimates were removed, we averaged their verbal estimates of 40° and 62° only.

**Fig. 2:**
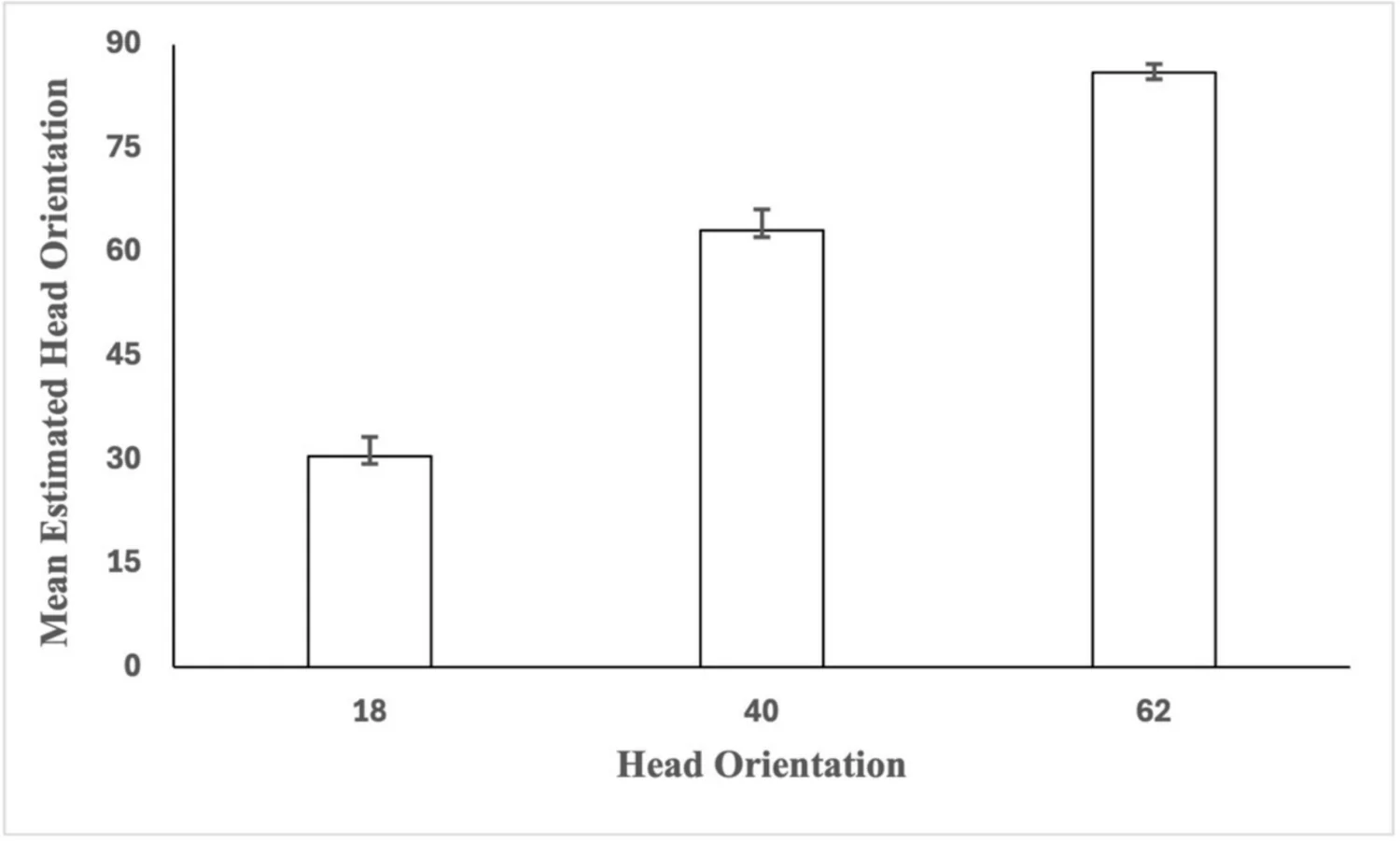
Actual head orientation versus estimated head orientation is shown. Error bars are also shown (standard errors)

Means and standard deviations for each orientation are shown in Table 1 and also shown graphically in Fig. 2. We converted observers’ verbal estimates of where their head was oriented versus where it was actually oriented to gains across the three angles they were asked to estimate (verbal estimate of head orientation/actual head orientation). To test whether observers overestimate their head orientation, we compared the values of their gains to a value of 1, which is what one would expect if observers’ estimates of head orientation were exactly equal to actual head orientation. A Bayesian one-sample *t*-test analyzing whether observers significantly overestimated their head orientation across all angles found that they did: *BF*_*10*_ = 5.19 × 10^6^ (decisive evidence), > Test value (one-tailed), *t*(29) = 8.21, *p* <.001, η^2^ = 0.36, *M* = 1.56 (*SD* = 0.37), 95% Credible Interval: {1.419, 1.698},^5^ Cauchy Prior with a scale of.707. This may be interpreted that it is more than 5.19 a 10^6^ times as likely that there is a difference between verbal estimates of upward head orientation and the actual orientation than that there is no difference. We then tested the gains to a value of 1.5, the factor by which observers overestimate the slope of virtual, man-made, and geographical slopes as well as the factor by which observers overestimate downward head orientation. A Bayesian one-sample *t*-test found that there was no statistical difference between observers’ upward head orientation gains and that of downward head orientation and verbal estimates of orientations of surfaces, *BF*_*01*_ = 3.67 – substantial evidence in favor of the null, *t(*29) = 0.86, *p* =.398, Cauchy Prior with a scale of.707. This may be interpreted as it being more than three times as likely that there is no difference between verbal estimates of upward head orientation and a value of 1.5 than there is a difference.

**Table 1:** Means and standard deviations for head orientation estimates of each of the orientation positions in Experiment 1a

Angle	Mean	*SD*
18°	30.43°	15.04°
40°	63.17°	16.16°
62°	85.97°	6.61°

#### Experiment 1b: Upward head orienting to verbally given angles (22°, 45°, 67°)

**Fig. 3:**
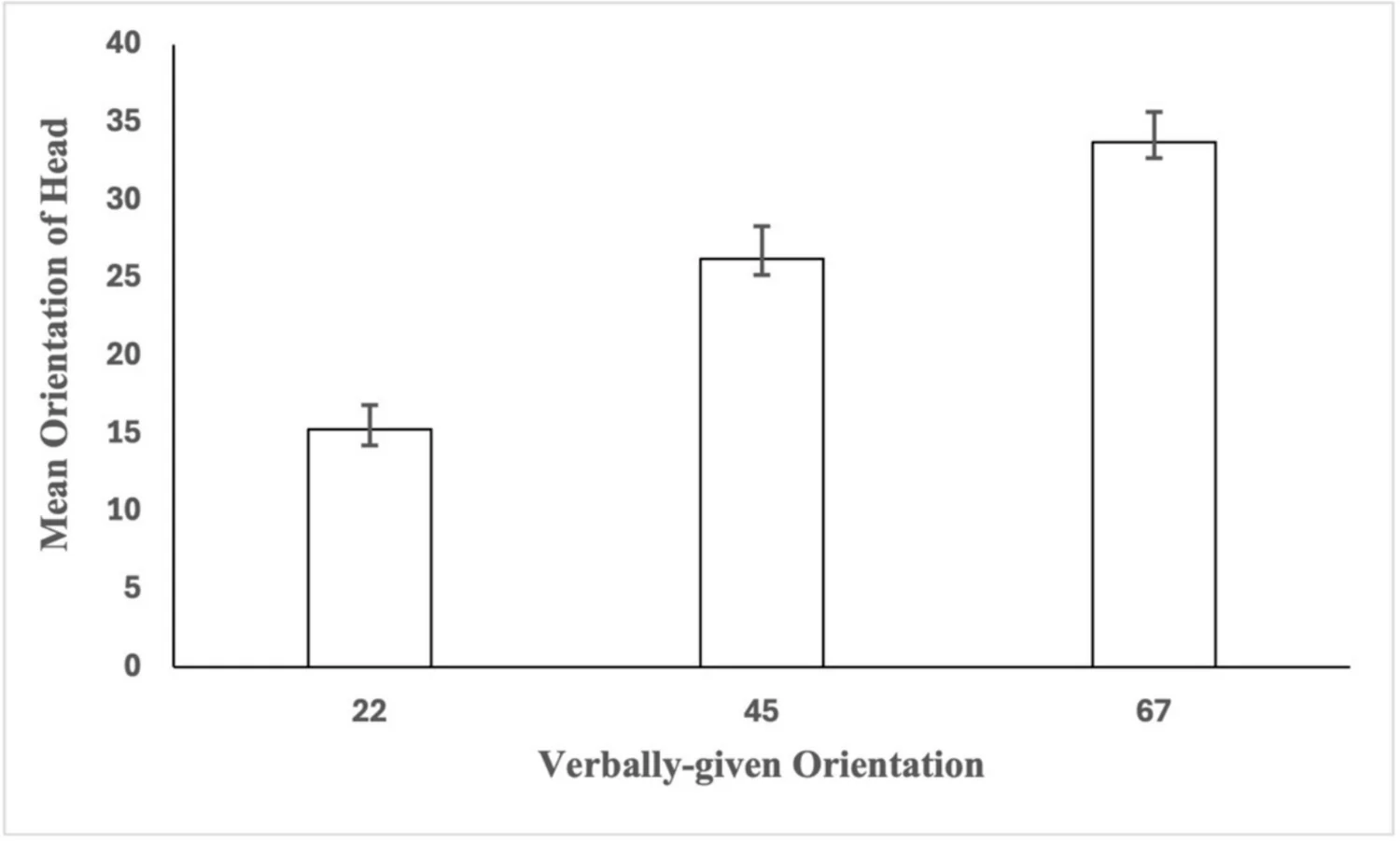
Verbally given head orientation versus where participants oriented their head orientation is shown. Error bars are also shown (standard errors)

No estimates of 18° exceeded 60° so no cases were removed. Means and standard deviations for each orientation are shown in Table 2 and Fig. 3. We converted observers’ positioning of their own head orientation to the verbally given orientation (head orientation/verbally given orientation) across the three verbally given angles. An overestimate of head orientation by a factor of 1.5 in upward head orientation would mean that if someone were asked to estimate their head orientation when we positioned their head at 30°, they would estimate it to be 45° (or their verbal estimate would be 1.5 times as great as the head orientation we asked them to estimate). Drawing from Experiment 1a, the mean gain across head orientations was 1.56, which means that if we oriented their head at 30°, they would estimate it to be ~46.8°. This also means that for what we asked of participants in the current experiment – asking them to orient their head at a verbally given angle – we should expect the inverse result. That is, if they overestimate upward head orientation by a factor of 1.56 as they did in Experiment 1a, when we ask them to orient their head at 30°, 30° should feel like 46.8° and their gain of where they orient their head should be 30°/46.8° =.64, much smaller than a value of 1. In order to test whether observers overestimate their gaze orientation, we compared the values of their gains to a value of 1, which is what one would expect if observers’ gaze orientation reproduction of verbally given angles were exactly equal to the verbally given angles. A Bayesian one-sample *t*-test analyzing whether observers significantly overestimated their upward gaze orientation (i.e., stopped their upward gaze orientation shallow of the verbally given orientation) across all verbally given angles found that they did: *BF*_*10*_ = 1.75 × 10^7^, < Test value (one-tailed), *t*(29) = −8.73, *p* <.001, η^2^ = 0.39, *M* =.5973 and *SD* =.253, 95% Credible Interval: {0.504, 0.692}, Cauchy Prior with a scale of.707. We then tested the gains to a value of.64, the value we would predict given the results of Experiment 1a. A Bayesian one-sample *t*-test comparing verbal estimate of gaze orientation to a value of 0.64 also found that there was no statistical difference between verbal estimates and the value of 0.64, *BF*_*01*_ = 3.54 (substantial evidence for no difference), *t(*29) = −0.91, *p* =.373, Cauchy Prior with a scale of.707. Interpretation of the Bayes factor indicates that it is almost four times as likely that there is no difference in reproduced orientations of gaze and a value of 0.64.

**Table 2:** Means and standard deviations for head orientation estimates of each of the requested orientations in Experiment 1b

Angle	Mean	*SD*
22°	15.27°	8.74°
45°	26.21°	11.51°
67°	33.72°	10.34°

#### Experiment 1c: Verbal estimates of downward head orientations (18°, 40°, 62°)

**Fig. 4:**
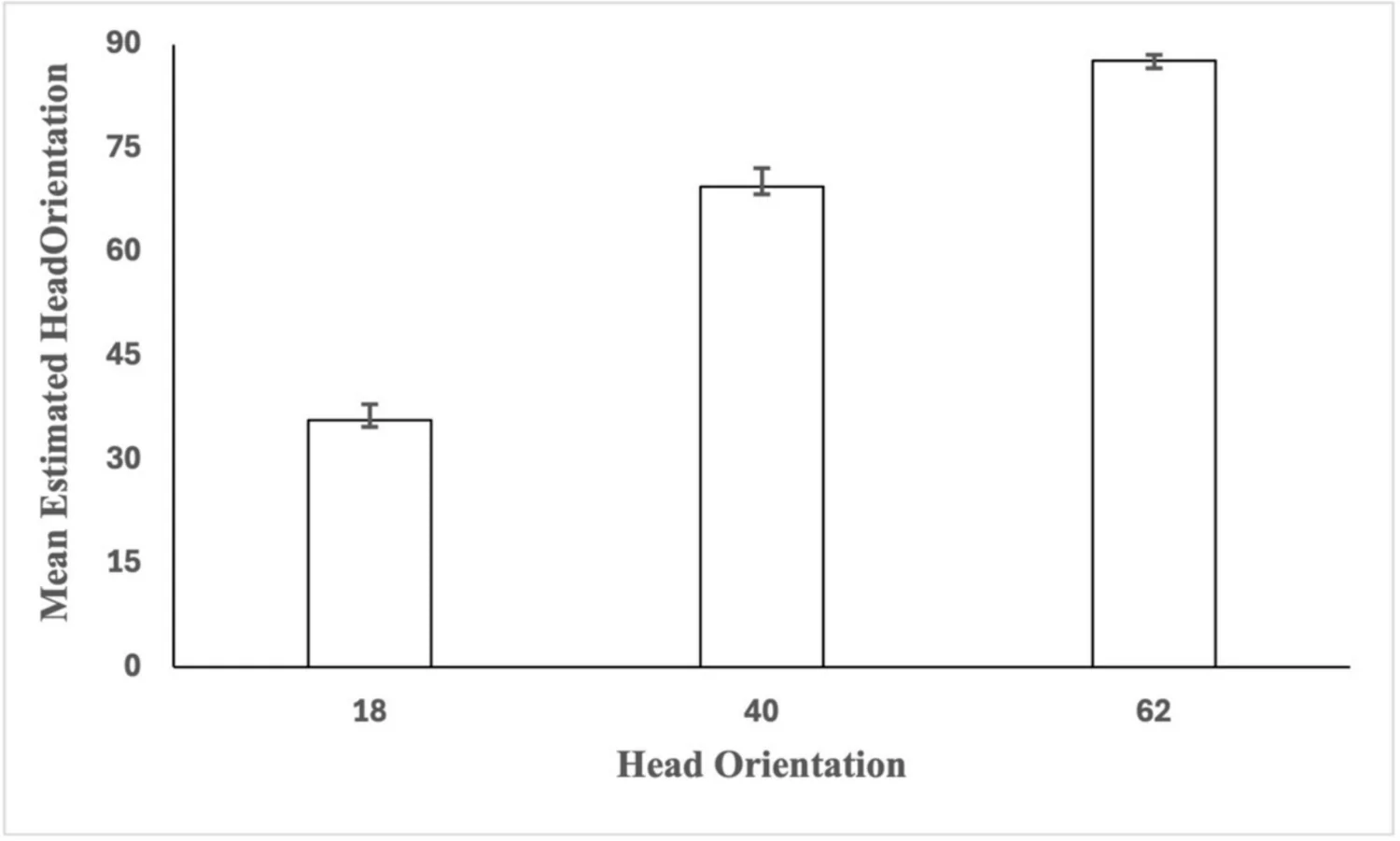
Actual head orientation versus estimated head orientation is shown. Error bars are also shown (standard errors)

Seven estimates of 18° equaled or exceeded 60° (60° (3), 65°, 70° (2), 75°). These estimates were removed. For those seven participants we averaged the verbal estimates of 40° and 62° only. Means and standard deviations are shown in Table 3 and Fig. 4. We converted observers’ verbal estimates of where their head was oriented versus where it was actually oriented to gains across the three angles they were asked to estimate (verbal estimate of head orientation/actual head orientation). To test whether observers overestimate their downward head orientation, we compared the values of their gains to a value of 1, which is what one would expect if observers’ estimates of downward head orientation were exactly equal to actual head orientation. A Bayesian one-sample *t*-test analyzing whether observers significantly overestimated their downward head orientation across all angles found that they did: *BF*_*10*_ = 5.59 × 10^11^, > Test value (one-tailed), *t*(29) = 13.89, *p* <.001, η^2^ = 0.62, *M* = 1.69, *SD* = 0.27, 95% Credible Interval: {1.591, 1.795}, Cauchy Prior with a scale of.707. We then tested whether there was a difference between downward head orientation overestimates and upward head orientation overestimates in Experiment 1a. A Bayesian one-sample *t*-test comparing verbal estimates of downward head orientation to the value of 1.56, the gain found in Experiment 1a for upward head orientation, also found that there was no statistical difference between verbal estimates and the value of 1.56, Bayes factor = 2.14 in favor of no difference *t*(28) = 1.4, *p* =.173, Cauchy Prior with a scale of.707.

**Table 3:** Means and standard deviations for head orientation estimates of each of the head positions in Experiment 1c

Angle	Mean	*SD*
18°	35.7°	11.21°
40°	69.4°	14.68°
62°	87.6°	5.16°

#### Experiment 1d: Verbal estimates of head orientation in the azimuth direction (18°, 40°, 62°)

**Fig. 5:**
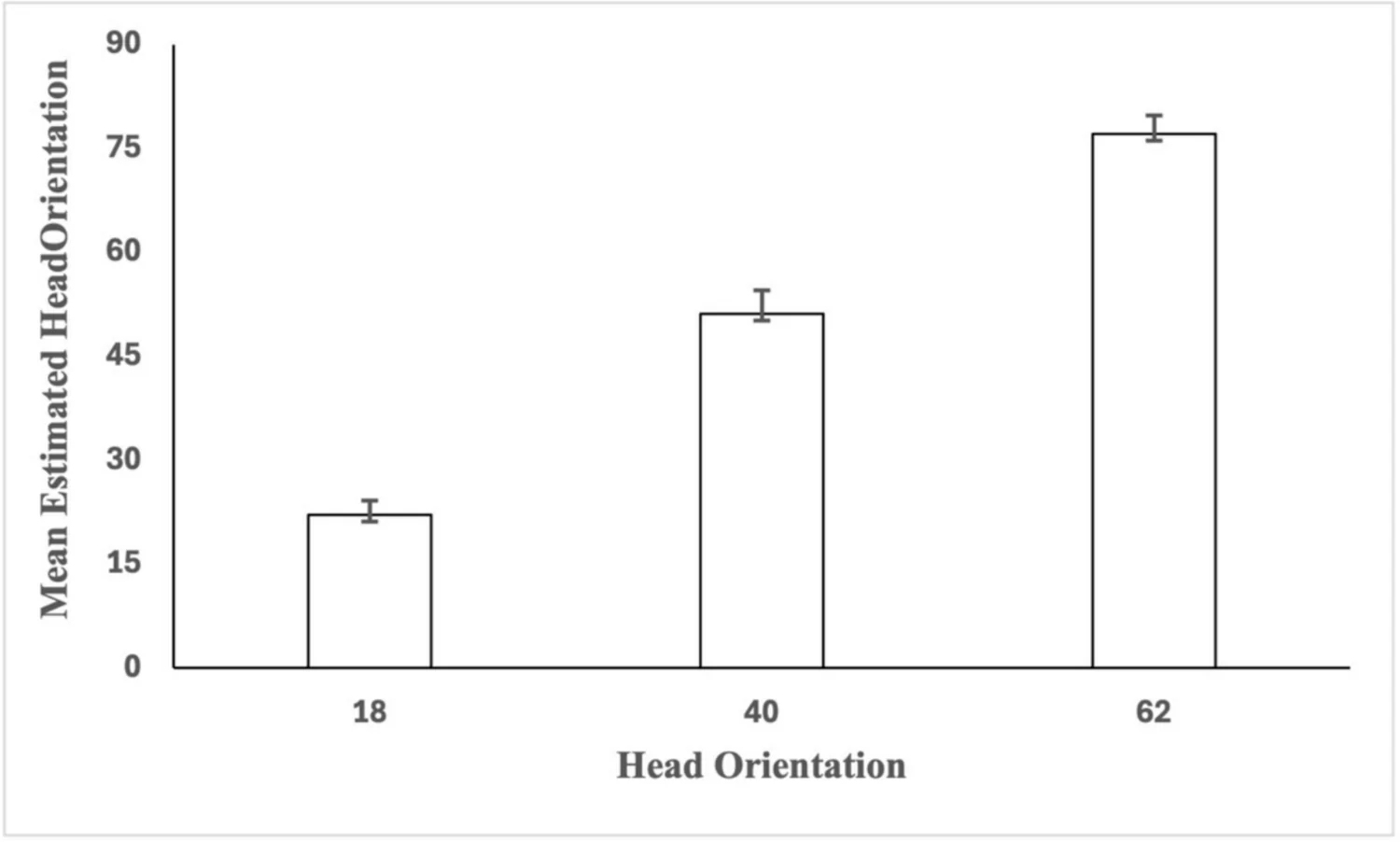
Actual head orientation versus estimated head orientation is shown. Error bars are also shown (standard errors)

Two estimates of 18° exceeded 60° (65° (2)). These estimates were removed. For those two participants we averaged their verbal estimates of 40° and 62° only. Means and standard deviations for each orientation are shown in Table 4 and Fig. 5. We converted observers’ verbal estimates of where their head was oriented versus where it was actually oriented to gains across the three angles they were asked to estimate (verbal estimate of head orientation/actual head orientation). We first tested whether there were differences in azimuth estimates for people turning left versus those turning right. A Bayesian independent-samples *t*-test showed no statistical difference in azimuth direction tested, *BF*_*01*_ = 1.74, *t*(28) = 1.17, *p* =.252, *M*_*Left*_ = 1.33, *SD*_*Left*_ =.27; *M*_*Right*_ = 1.18, *SD*_*Right*_ =.4. To test whether observers overestimate their azimuth head orientation, we compared the values of their gains to a value of 1, which is what one would expect if observers’ estimates of head orientation were exactly equal to actual head orientation. A one-sample *t-*test analyzing whether observers significantly overestimated their azimuth head orientation across all angles found that they did: *BF*_*10*_ = 178.16, *t*(29) = 4.07, *p* <.001, η^2^ = 0.12, *M* = 1.254 (*SD* = 0.341), 95% Credible Interval: {1.126, 1.381}, Cauchy Prior with a scale of.707. This gain is virtually identical to 1.26, the gain found for azimuth head estimates by Li and Durgin (2016).

**Table 4:** Means and standard deviations for head orientation estimates of each of the head positions in Experiment 1d

Angle	Mean	*SD*
18°	22.14°	10.56°
40°	51.17°	17.96°
62°	77.13°	14.6°

### Discussion

We have found that observers overestimate upward head orientation similarly to how observers overestimate downward head orientation as well to how observers overestimate the surface orientation of man-made, virtual, and geographical hills (Durgin & Li, 2011; Li & Durgin, 2009; Proffitt et al., 1995; Shaffer & Flint, 2011; Shaffer et al., 2019; Shaffer & McManama, 2015). This was shown both by participants’ verbal estimates to different head orientations as well as by their orienting their head to a verbally given orientation. While the gains were different in Experiments 1a and 1b, we used two different methods. In Experiment 1a, we asked participants to verbally estimate their head orientation. Therefore, overestimating their head orientation would mean that their estimate should be greater than their actual head orientation. In Experiment 1b, we asked participants to reproduce a verbally given orientation. Therefore, overestimating their head orientation would mean that their head orientation should be less than the verbally given orientation. For instance, say observers overestimate vertical head orientation by a factor of 1.56, which they did in Experiment 1a. Therefore, if they are verbally overestimating their head orientation, when asked to verbally estimate their head orientation when their head is tilted upward at 30°, they would give a verbal estimate ~ 47° and their gain would be 47°/30° ~ 1.56; if they are asked to orient their head at ~47°, they should orient their head at ~30° as 30° feels like 47° and their gain would be 30°/47° or ~ 0.638 (it was.5973 in Experiment 1b) (Shaffer et al., 2016a, b).

Overestimation of perceived upward head orientation found in Experiment 1a was statistically equivalent to perceived downward head orientation found in Experiment 1c. The factor by which participants overestimated upward head orientation is not only similar to that given when people estimate the orientation of man-made, virtual, and geographical slopes, but also similar to the factors by which people overestimate their own hand orientation (1.63 and 1.66, Shaffer et al., 2016a, Experiments 2 and 3, respectively) and by which people overestimate their own body tilt backward (1.457, Shaffer et al., 2016b). This extends the findings of downward head orientation to upward head orientation and shows that head orientation in the sagittal plane (or elevation coordinate) crosses the transverse plane leading out from an upright person’s head and eyes, and in general is misperceived in the same way.

Downward head orientation was overestimated slightly more than shown by others previously (1.69 in Experiment 1c compared to ~1.5; Durgin & Li, 2011; Li & Durgin, 2009). This may have been due to the added weight of the helmet and magnetic angle locator. However, Li and Durgin (2009) showed that proprioception of downward head orientation with a limited view could be overestimated by a factor of up to 2 (Experiment 2B). Given this, it may be that perception of head orientation rather than gaze orientation may lead to greater overestimates. However, across a range of slopes Li and Durgin (2009) found that the best-fitting gain factor was 1.54 (Experiment 3A). In light of this, it seems that the downward head overestimates shown in the current work fit rather well with what has been previously shown.

Head orientation in the azimuth coordinate was overestimated similarly to what has been found previously (Li & Durgin, 2016). This work extends that work by comparing explicit verbal orientation estimates in the azimuth coordinate to those in the elevation coordinate within the same participants. This not only confirms previous work but also gives us reliable indicators of how people mis(perceive) explicitly measured head orientation in all three directions.

## Experiment 2a and b

Experiment 2a investigated a shallower range of upward head orientations than were used in Experiment 1a. Li et al. (2011) estimate that the relevant range of head declinations is probably less than 50°–60°. This fits with other work showing that orientations of the head (say here, α) during normal everyday activities is: −50° < α < +50° (Sinnot et al., 2023). Since two of our angles (42° and 60°) in our original range of angles tested in the upward direction were close to or above this relevant range within which scale expansion is typically reported (Durgin et al., 2010; Sinnot et al., 2023), in Experiment 2a we asked participants to verbally estimate upward head orientations of 9°, 18°, and 27°.

In Experiment 2b, we investigated the range of upward and downward head orientations that people perceived as being straight up (90° upward), straight down (90° downward), and straight ahead (0°, with the line of gaze parallel to the ground). Perceptual ranges seem to occur with gaze/head orientations in different gaze/head directions and also seem to occur with the orientations of slanted surfaces. For instance, downward gaze orientation scale expansion seems to occur with angles reaching around 50° or so (Durgin et al., 2010). Similarly, Li and Durgin (2016) examining azimuth overestimation found a gain ~1.26 and across their study and three others found a linear fit with a gain of 1.22 out to eccentricities up to 40°. Scale expansion with gains of 1.5 have been found in previous work for slanted surfaces up to ~50° and scale contraction with slanted surfaces steeper than that (> 60°) (Durgin & Li, 2011; Durgin et al., 2010). It has also been shown that we are more sensitive to differences in slanted surfaces at the lower end of the range (Durgin & Li, 2011; Proffitt et al., 1995; Ross, 1994). Additionally, experts at climbing hills cannot discriminate between hills of ~72° and 90° and hills greater than 60° are unclimbable without special equipment, so it makes sense that we overestimate slanted surfaces by a factor of 1.5 (so a 60° hill should appear as if it is 90°) for at least two reasons. First, a scale expansion by a factor of 1.5 should make a 60° hill appear as if it is 90°, and second and more practically, if a 60° is completely unclimbable, then why should we even approach it thinking we might want to/be able to climb it? Further evidence showing that the perceptual range of upward head orientations might be compressed is that in Experiment 1a, ~83% of estimates were at or above 85° for the 62° angle, and almost 57% of estimates were 90° (downward head estimates showed the same modal response of 90° for the 62° angle and the 90° estimate comprised one-third of the estimates).

We imagine just like with perceived downward gaze and head orientation as well as with perceived slanted surfaces, there is a range within ± 90°, which people are able to, and more likely use functionally and scale their perception of the spatial layout of the environment within a more compressed area of this ± 90°. The restricted range of head orientations has, to our knowledge, never been studied before, and should provide us with some kind of blueprint of ranges within which people are able to and most often utilize.

### Method

#### Participants

Thirty-three participants (24 female) whose mean age was 19.06 years (*SD* = 1.78) who were independent of those in Experiment 1 from the Ohio State University at Mansfield participated in fulfillment of an Introductory Psychology requirement. Each participant was required to sign a consent form for the study. Informed consent was obtained for all participants. This study was approved by The Ohio State University Behavioral and Social Sciences Institutional Review Board (Study Number: 2023B0282).

#### Power analyses

The first reason we used *n* = 33 participants in this study is discussed was discussed in the *Method* section of Experiment 1 and is based on previous related work.

As we did in Experiment 1, we planned to perform a one-sample *t*-test for comparing an index of participants’ overestimations to a value of 1 (Experiment 2a) and either 90° (two conditions) or 0° (third condition), indicating veridical perception for each comparison. In two experiments that closely match not only the methodology and focus of this study – having people verbally estimate different orientations or reproduce orientations to a verbally given angle (Shaffer et al, 2016a, Experiments 1 and 3), but also the analysis – using one-sample *t*-tests to analyze estimates compared to actual orientations, 30 participants were used in each experiment. In both of these experiments statistically significant effects were found. In order to determine the proper number of participants, we used the results of Shaffer et al. (2016a) as a guide for the effect size to use for our preliminary analysis. In those experiments, effect sizes of Cohen’s* d* =.18,.57,.97, 1.39, 1.22 were found. We took the mean Cohen’s* d* across those experiments and found an average effect size of Cohen’s* d* =.866. Using a one-tailed test, participants will overestimate (Experiment 2a, 0° condition of Experiment 2b) or underestimate (90° conditions of Experiment 2b) the actual head orientation, an effect size of *d* =.866, *α* =.05, Power =.95, the total sample size necessary would be *n* = 16. For this power analysis we used G*Power 3.1.9.6 (Faul et al., 2007) and for the design we used the “*t*-tests: Means: Difference from constant (one-sample case)” procedure and the Type of power analysis was “A priori: Compute required sample size – given *α* power, and effect size.”

#### Sensitivity power analyses

Consistent with the recommendations of Giner-Sorolla et al., (2024) and Lakens (2022), once we decided on a sample size of 33 due to previous studies and the aforementioned a priori analysis, we performed an effect size sensitivity analysis in order to determine the minimal effect it would take for this analysis to detect a difference between an index of participants’ overestimates (Experiment 2a and the 0° condition of Experiment 2b) compared to a value of 1 or 0° (veridical perception) or underestimates (90° conditions of Experiment 2b) and a value of 90° (veridical perception), respectively. This analysis had sensitivity power using a one-tailed test participants would verbally overestimate (Experiments 2a and the 0° condition of Experiment 2b), or underestimate orientations (90° conditions of Experiment 2b), *α* =.05, Power =.95, and *n* = 33, the minimal effect our study would detect is *d* =.59 (effect sizes of *d* =.44 and.52 can be detected for Power = 80% and 90%, respectively (Lakens, 2022)). For this power analysis we used G*Power 3.1.9.6 (Faul et al., 2007) and for the design we used the “*t*-tests: Means: Difference from constant (one-sample case)” procedure, and the Type of power analysis was “Sensitivity: Compute required effect size – given *α*, power, and sample size.”

#### Design

In Experiment 2a, with participants seated in the laboratory in a chair that allowed them to freely move their head, neck, and shoulders, researchers oriented participants’ head direction at three different orientations (9°, 18°, and 27°) in the upward direction and participants verbally estimated their head orientation. We randomized the order of the three angles. In Experiment 2b, again with participants seated in the laboratory, participants oriented their head upward at 90°, downward at 90°, and at 0°. We randomized the order in which they did this. In previous work, it has been shown that are able to report perceived gaze and head orientation (Durgin & Li, 2011; Li & Durgin, 2009).

#### Materials

A magnetic angle locator was secured on a bicycle helmet that participants wore to measure head orientation. When looking in front, many participants looked upward enough to be looking at an all-white ceiling. When looking downward, many participants looked downward far enough to be looking at an all light blue carpet on the floor.

#### Procedure

All participants took part in both conditions. The order of the conditions was randomized for each participant as were the orientations used within each condition. Participants were first seated in a laboratory. We then secured a bicycle helmet to their head. In Experiment 2a, after the researcher moved their head to a given orientation, we recorded their verbal estimate of the angle, and then the researcher moved their head to the subsequent orientation. In Experiment 2b, after the participant moved their head to the requested orientation the participant paused, we recorded the angle in degrees on the magnetic angle locator on the helmet, and then participants moved their head to the subsequent orientation. They did this until they estimated all six orientations across both conditions. We randomized the order of the orientations and had them estimate a subsequent orientation from the position of the previous orientation instead of having them start from the same orientation to avoid anchoring biases (Shaffer et al., 2014, 2015). We also told participants to look straight ahead with their eyes as their head moved. We stationed a researcher in front of them to make sure they were doing this.

### Results

For Experiment 2 and prior to analyzing the data, we decided to remove any estimates of 9° that equaled or exceeded 45° as that would show a clear misunderstanding of angles in general and not necessarily reflect a perceived feeling of head orientation as being greater than actual. We also removed any verbal estimates where the angle of 18° was estimated at or greater than 60° (as we did in Experiments 1a, 1c, and 1d) or where the angle of 27° was estimated at 90°. This is because some of the aforementioned work has shown gains of 1.5 and that climbers cannot distinguish between hills of ~60° to 90°. This means that someone who had their head oriented at and gave a verbal estimate of greater than 60° in any direction when their head orientation was at 18° would essentially perceive their head was tilted maximally. Also given that all participants were instructed as to what 0° and 90° is and the restrictions of the head and neck (especially in upward and downward directions), it would seem that estimates of 18° that equaled or exceeded 60° or estimates of 27° that were equivalent to 90° are not as much overestimates of head orientations as they are a misunderstanding of what they were asked to do. If that happened, we simply removed that estimate but kept the remaining estimates of the other two angles for that participant. For these cases, if these data restrictions removed two of their three estimates, we removed all of that participant’s estimates from the analyses.

#### Experiment 2a: Verbal estimates of upward head orientations (9°, 18°, 27°)

**Fig. 6:**
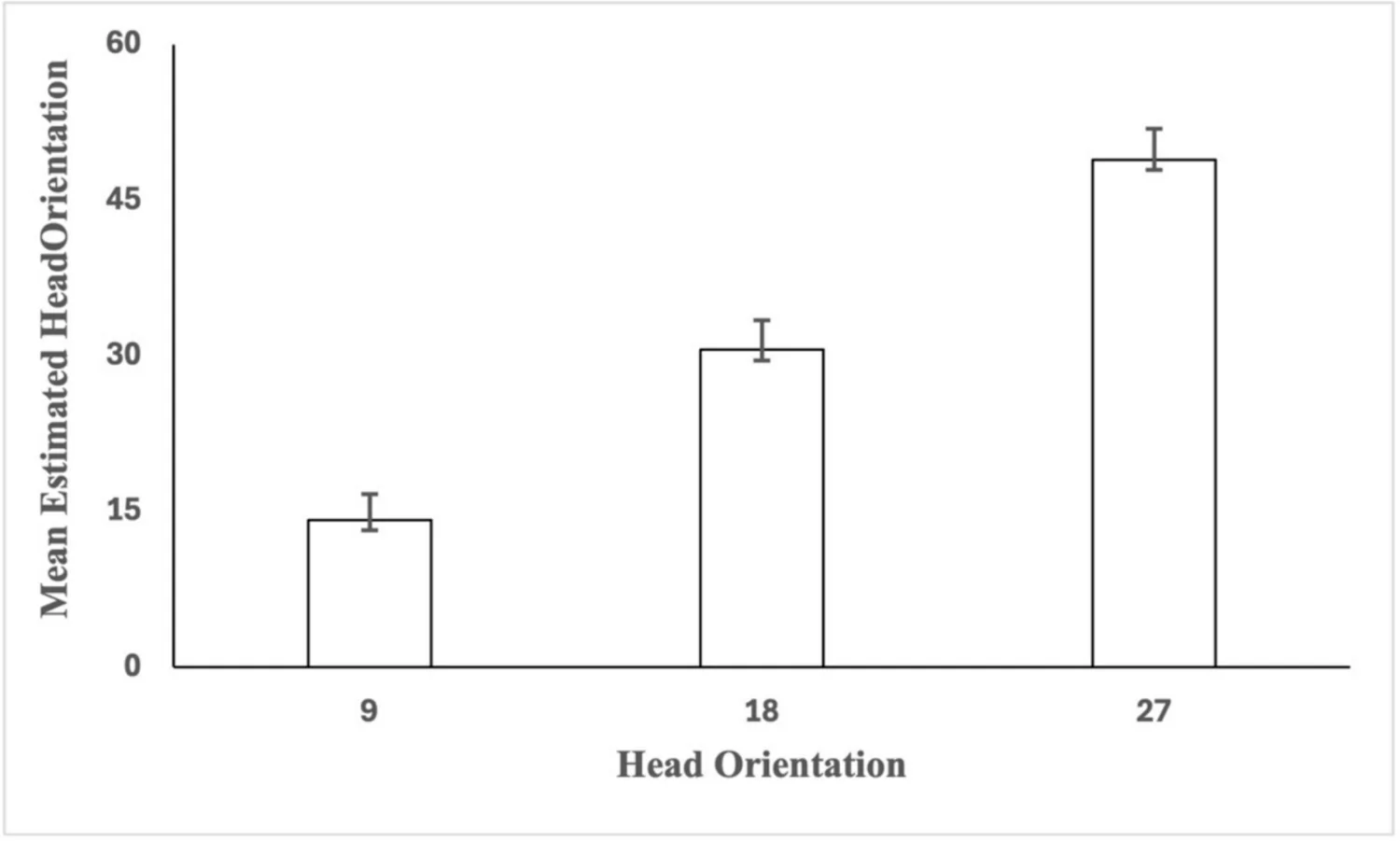
Actual head orientation versus estimated head orientation is shown. Error bars are also shown (standard errors)

Seven participants’ estimates of 9° and 18° equaled or exceeded (45° and 60°, respectively – ranges from 45° to 75° for 9° (45°, 55°, 60°, 70°, 75° (2), 80°) and 60° to 90° for 18° (65°, 75° (2), 80° (2), 85° (2)). One participant’s estimates of 18° and 27° were both 90°. So we removed those eight participants’ data from the analyses. One participant’s estimates for 18° exceeded 60° (70°) and one participant’s estimate of 9° was 45°, so we removed their estimates of 9° and 18°, respectively, and averaged across their remaining estimates. Means and standard deviations for each orientation are shown in Table 5 and Fig. 6. To test whether observers verbal overestimate their head orientation, we compared the values of their gains to a value of 1, which is what one would expect if observers’ estimates of head orientation were exactly equal to actual head orientation. A Bayesian one-sample *t*-test analyzing whether observers significantly overestimated their head orientation across all angles found that they did: *BF*_*10*_ = 916.76. > Test value (one-tailed), *t*(24) = 11.84, *p* <.001, η^2^ = 0.58, *M* = 1.7 (*SD* = 0.72), 95% Credible Interval: {1.406, 2.001}, Cauchy Prior with a scale of.707. We then tested the gains to a value of 1.5, the factor by which observers overestimate the slope of virtual, man-made, and geographical slopes as well as the factor by which observers overestimate downward gaze orientation. A Bayesian one-sample *t*-test found that there was no statistical difference between observers’ gains in the elevation coordinate that that of verbal estimates of orientations of surfaces or their downward gaze, *BF*_*01*_ = 1.96, *t(*24) = 1.41, *p* =.17, Cauchy Prior with a scale of.707.

**Table 5:** Means and standard deviations for head orientation estimates of each of the head positions in Experiment 2a

Angle	Mean	*SD*
9°	14.21°	10.34°
18°	30.58°	13.72°
27°	48.91°	14.3°

#### Experiment 2b: Head orientations to 90° upward, 90° downward, and 0° (straight ahead)

**Fig. 7:**
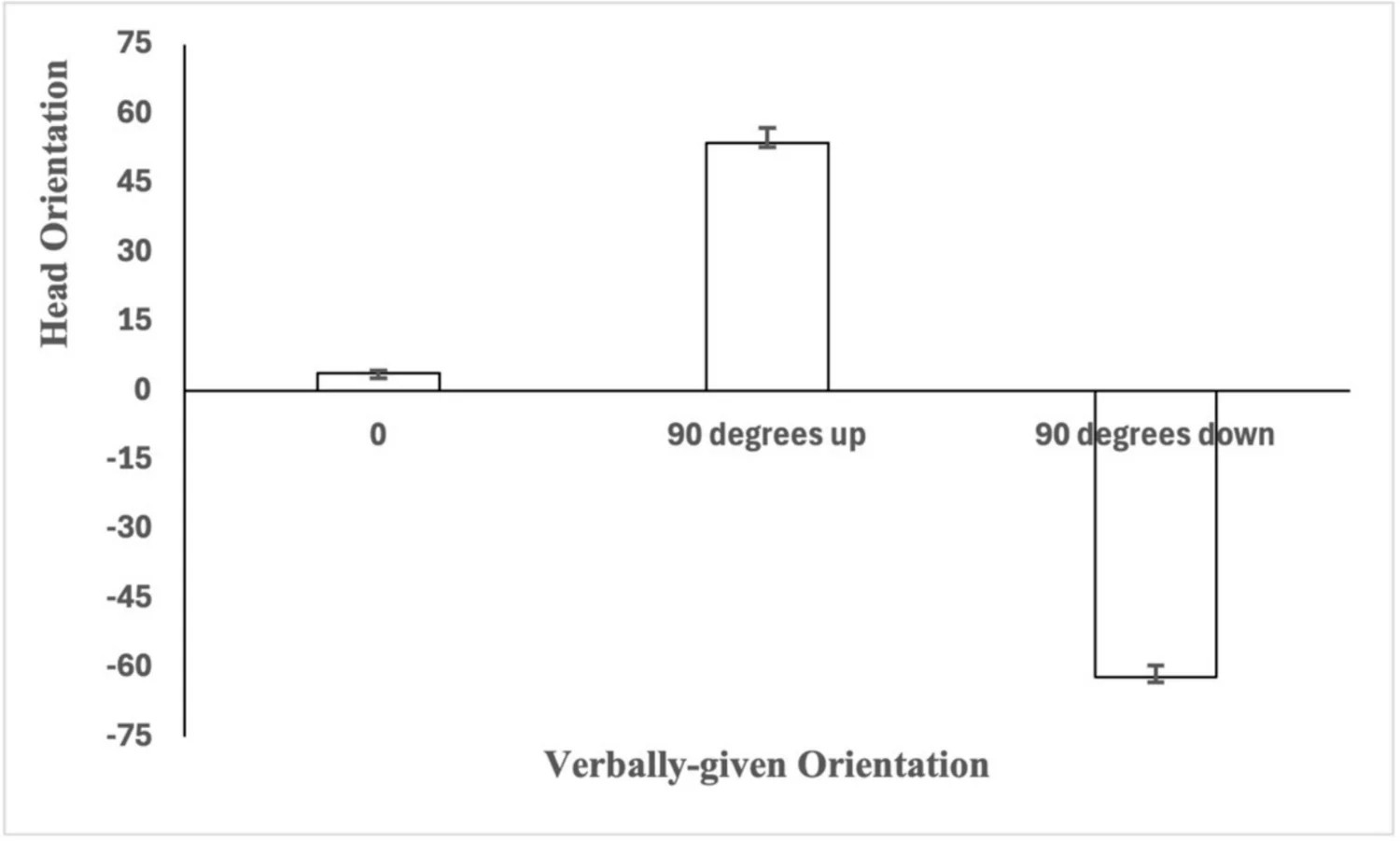
Verbally given head orientation versus where participants oriented their head orientation is shown. Error bars are also shown (standard errors)

No restrictions were placed on the data, so all 33 participants are included in the analyses. Means and standard deviations are shown in Table 6 and Fig. 7. When asked to orient their head orientation at 90° upward, participants set their head orientation at 53.64° A Bayesian one-sample *t*-test analyzing whether observers set their head orientation to significantly less than 90° found that they did, *BF*_*10*_ = 1.49 × 10^10^, < Test value (one-tailed), *t*(32) = −11.28, *p* <.001, *η*^*2*^ = 0.49, 95% Credible Interval: {47.08°, 60.2°}, Cauchy Prior with a scale of.707. When asked to orient their head at 90° downward, participants oriented their head at 62.08°. A Bayesian one-sample *t*-test analyzing whether observers oriented their head to significantly less than 90° found that they did, *BF*_*10*_ = 7.95 × 10^9^, < Test value (one-tailed), *t*(32) = −10.997, *p* <.001, *η*^*2*^ = 0.48, 95% Credible Interval: {56.91°, 67.25°}, Cauchy Prior with a scale of.707. A Bayesian one-sample *t*-test analyzing whether observers oriented their head to significantly more than 0° found that the average orientation at which they oriented their head was 3.77°, *BF*_*10*_ = 151,352.97, > Test value (one-tailed), *t*(32) = 6.58, *p* <.001, *η*^*2*^ = 0.25, 95% Credible Interval: {2.599°, 4.931°}, Cauchy Prior with a scale of.707.

**Table 6:** Means and standard deviations for head orientation estimates of each of the orientation positions in Experiment 2b

Angle	Mean	*SD*
90° Up	53.64°	18.51°
0° (Straight Ahead)	3.77°	3.29°
90° Down	62.08°	14.59°

### Discussion

Experiment 2 first showed that similar overestimations of upward orientation occur at a shallower range of angles (9°–27°) as they do at a steeper range of angles (18°–62°). The shallower range of angles had a slightly higher gain (1.7) than the steeper range (1.56) though not statistically so. Experiment 2 also showed that people’s perceived maximum head orientation at 90° upward and downward as much less steep than actual, with means of 53.64° and 62.08°, respectively. Restricted ranges of gaze orientation have been previously reported (Durgin & Li, 2011; Durgin et al., 2010; Li et al., 2011). For instance, Li and Durgin (2009) asked observers to estimate gaze declination of objects in a natural environment and objects with an orientation of 43.3° produced perceived gaze declinations of ~60°–70° for two-dimensional orientation matches and verbal estimates. This fits with the research that shows the factor by which observers overestimate sloped surfaces is ~1.5 up to sloped surfaces < 50°, and the research that shows how observers are more sensitive to differences in slanted surfaces at the lower end of the range (Durgin & Li, 2011; Durgin et al., 2010; Li et al., 2011; Proffitt et al., 1995; Ross, 1994). This also seems to be of functional use with hills greater than 60° being unclimbable without special equipment and it being difficult to distinguish from ~60° to 90° hills among expert climbers (Proffitt et al., 1995; Ross, 1994). Our work here supports this. In fact, when we multiply the estimates of 90° from Experiment 2b by the average gain of estimates from Experiment 2a, the mean is not statistically different from 90°, *BF*_*01*_ = 5.25 *t*(32) =.22, *p* =.83, *M* = 91.19°, *SD* = 31.47°, Cauchy Prior =.707. So it seems like participants scaled their perceived upward head orientation to match ~0° to 90° coordinate system.

The head orientation inclination and declination boundaries are no doubt due, at least in part, to the musculature and skeletal restrictions of the head and neck to accommodate looking “straight down” or “straight up” in addition to the movement of the eye within its orbit and occlusion (at least in the upward direction) by the eyelids. Restrictions like this have been reported for hand movements made to reproduce the orientation of sloped surfaces due to similar restrictions of movements of the hand and wrist with respect to the forearm (Shaffer et al., 2016a). Regarding upward head/gaze orientation, it seems that there are few situations where one would need a large perceived range of head/gaze orientations. For instance, when looking out of windows in one’s house or the windshield of one’s vehicle, if would be rare if your gaze would be inclined at more than 25°–30° while sitting or standing looking out of them. Even in the rarer situations where gaze must be inclined a great deal, as is the case with outfielders in baseball catching fly balls, players heading a ball in the world’s football, dogs and people chasing Frisbees, and receivers in American football catching passes, gaze inclination typically is in the range of 20°–50° and rarely exceeds 60° (McBeath et al., 1995; McLeod et al., 2008; Shaffer, et al. 2004, 2013; Shaffer & McBeath, 2005; Shaffer et al., 2008a, b). One would expect a wider range of movement in the azimuth direction due to fewer musculature and skeletal restrictions of head and neck. While we did not measure azimuth head orientation boundaries in the current work, if the gains reflect how we scale the world and the perceptual boundaries of gaze and head orientation, then the gains of 1.26 found in the current and precious work certainly suggests a wider range of movement to perceived outer boundaries in the azimuth direction. This also matches previous work showing that the azimuth direction is overestimated less than upward and downward gaze orientations and fits with the idea that the range might be scale expanded so that maximum estimates are equivalent to a 90° range as they were here (Durgin & Li, 2011; Li & Durgin, 2016).

In the current work, we have measured head orientation with eyes straight forward in line with gaze. We had participants keep their eyes open the whole time while measuring head orientation. While much of the background at which participants were looking was of uniform texture (white, with the exception of the all light blue carpet for the many participants who looked in the downward direction far enough to see it), depending on where they looked, the background wall had well-defined separations from ceiling and floor. While we designed the study and the instructions to the participants to focus on where their head was aiming (more proximally), we have no way of knowing how much at what they were looking at played a role in their head orientations or verbal estimates of head orientation. This is the first reason we decided to conduct Experiment 3. The second reason is that Experiments 1 and 2 measured head orientation with eyes straight forward. Because their eyes were open, this was not exactly the “purest” measure of head orientation, at least not independent of all other factors, including visual inputs. Hajnal et al. (2011) found differences between haptic slope estimates and visual estimates for their largest slope (16 $^\circ$) for slopes on which participants stood with non-informative vision (where they were not looking at the slope on which they were standing), and in a blindfolded condition. Shaffer et al. (2016b) found no differences in eyes open versus eyes closed condition when participants were estimating their own body tilt. However, while visual inputs certainly could contribute to proprioception in slope perception while standing on the slope and in body tilt while the body is being tilted, but certainly no more so than when measuring head orientation and its contribution to proprioception. Therefore, in Experiment 3, we measured head orientation independent of the integration with vision.

## Experiments 3a, 3b, 3c, and 3d

### Method

#### Participants

Twenty-four participants (17 female) whose mean age was 18.88 years (*SD* = 2.09) from the Ohio State University at Mansfield participated in fulfillment of an Introductory Psychology requirement. These participants were all different from those who participated in Experiments 1 and 2. Each participant was required to sign a consent form for the study. Informed consent was obtained for all participants. This study was approved by The Ohio State University Behavioral and Social Sciences Institutional Review Board (Study Number: 2023B0282).

#### Power analyses

Two factors informed our decision as to the number of participants to use in Experiment 3 (Lakens, 2022). First, 24 participants were used in this Experiment based on previous studies performed that most closely resemble the approach and methods of these studies – having participants estimate their gaze orientations – and also statistically used, at least in part, a repeated-measures design. Participants used in each of these studies were 35 (Li & Durgin, 2016, Experiment 1), 19 (Li & Durgin, 2016, Experiment 2), 13 (Li & Durgin, 2009, Experiment 2B), and eight (Li & Durgin, 2009, Experiment 3B) and 30 and 33 (in Experiments 1 and 2 of the current article) (Total* N* = 138/6 studies = 23). In all of these experiments statistically significant effects were found.

Second, in our analyses, we first planned to perform a one-way repeated-measures ANOVA (see Footnote 1). In order to determine the proper number of participants, we used the results of Li and Durgin (2016, Experiment 1) as a guide for the effect size to use for our preliminary analysis. In that work they used three different gaze conditions, found an *F* = 7.9 with *n* = 35. We converted these results to Cohen’s* f* =.4395 (see Footnote 2). Using an effect size of *f* =.4395, *α* =.05, Power =.95, one group with four measurements, correlation among repeated measures = 0.50, and a nonsphericity correction* α* = 1, the total sample size necessary would be *n* = 13. For this power analysis we used G*Power 3.1.9.6 (Faul et al., 2007) and for the design we used the “*F-*tests: ANOVA: Repeated-measures, within-factors” procedure and the Type of power analysis was “A priori: Compute required sample size – given *α* power, and effect size.”

For each condition, we also planned to perform a one-sample *t*-test for comparing an index of participants’ overestimations to a value of 1, indicating veridical perception for each condition. Participants used in three experiments that closely match not only the methodology and focus of this study – having people verbally estimate different orientation of their hand or head or orient them to a verbally given angle (Shaffer et al., current article, Experiment 1; Shaffer et al., 2016a, Experiments 1 and 3), but also the analysis – using one-sample *t*-tests to analyze estimates compared to actual orientations, was 30 in each experiment. In all of these experiments statistically significant effects were found. In order to determine the proper number of participants to use, we used the results of Shaffer et al. (2016a) as a guide for the effect size to use for our preliminary analysis. In those experiments, effect sizes of Cohen’s* d* =.18,.57,.97, 1.39, 1.22 were found. We took the mean Cohen’s* d* across those experiments and found an average effect size of Cohen’s* d* =.866. Using a one-tailed test (participants will overestimate (Experiments 3a–e) or underestimate (Experiment 3e, the 90° conditions) the actual gaze orientation, an effect size of *d* =.866, *α* =.05, Power =.95, the total sample size necessary would be *n* = 16. For this power analysis we used G*Power 3.1.9.6 (Faul et al., 2007) and for the design we used the “*t*-tests: Means: Difference from constant (one-sample case)” procedure and the Type of power analysis was “A priori: Compute required sample size – given *α* power, and effect size.”

#### Sensitivity power analyses

Consistent with the recommendations of Giner-Sorolla et al., (2024) and Lakens (2022), once we decided on a sample size of 24 due to previous studies and the aforementioned a priori analysis, we performed an effect size sensitivity analysis in order to determine the minimal effect it would take to detect a difference among the four conditions. Using *α* =.05, Power =.95, *n* = 24, one group with four measurements, correlation among repeated measures = 0.50, and a nonsphericity correction *ε* = 1, the minimal effect our study would detect is *f* =.308 (effect sizes of *f* =.245 and.28 can be detected for Power = 80% and 90%, respectively (Lakens, 2022)).^3^ For this power analysis we used G*Power 3.1.9.6 (Faul et al., 2007) and for the design we used the “*F*-tests: ANOVA, repeated-measures, within-factors” procedure. The following sections describe the analyses performed condition-by-condition.

We performed a second effect size sensitivity analysis in order to determine the minimal effect it would take for this analysis to detect a difference between an index of participants’ overestimates and a value of 1 (veridical perception). This analysis had sensitivity power using a one-tailed test (participants would verbally overestimate/underestimate orientations), *α* =.05, Power =.95, and *n* = 24, the minimal effect our study would detect is *d* =.692 (effect sizes of *d* =.523 and.616 can be detected for Power = 80% and 90%, respectively (Lakens, 2022)). For this power analysis we used G*Power 3.1.9.6 (Faul et al., 2007) and for the design we used the “*t*-tests: Means: Difference from constant (one-sample case)” procedure.

#### Design

Experiments 3a–d were composed of four within-subjects conditions. One change from the previous two experiments concerned the angles used. We decided to use angles of 15°, 33°, and 51° in Experiment 3 for two reasons. First, the results of Experiment 2 showed that many people believe their head orientation is perfectly horizontal when it is tilted upward at ~4° so we wanted to have a minimum angle than was greater than that was used in Experiment 2 (9°). Second, in Experiment 1, many participants gave estimates equal to or very close to 90° for a 62° angle. Therefore we moved our minimum and maximum angles farther away from 0° and 90° when participants were giving verbal estimates of head orientation, while still creating a large enough range within normal everyday head orientations (Sinnot et al., 2023). In Experiment 3a, researchers oriented participants’ gaze at three different orientations (15°, 33°, and 51°) in the upward direction and participants verbally estimated their gaze orientation. In Experiment 3b, participants were verbally given three angles at which to orient their gaze in the upward direction (22°, 45°, and 67°). In Experiment 3c, researchers oriented participants’ gaze at three different orientations (15°, 33°, and 51°) in the downward direction and participants verbally estimated their gaze orientation, and in Experiment 3d, researchers oriented participants’ gaze at three different orientations (15°, 33°, and 51°) in the azimuth (left or right) direction. We randomly assigned participants to either gaze to the left or gaze to the right for all three gaze orientations.

#### Materials

A digital magnetic angle locator was secured on a bicycle helmet that participants wore to measure head orientation for Experiments 3a–c. A magnetic angle inclinometer was secured on a bicycle helmet that participants wore to measure head orientation for Experiment 3d. Participants all wore a 100% light-blocking adjustable Manta Sleep™ mask.

#### Procedure

All participants took part in every condition. This was done to more directly compare upward, azimuth, and downward orientation perception. The order of the four conditions was randomized for each participant as were the three angles used within each condition. Participants were first seated in a laboratory in a chair that allowed them to freely move their head, neck, and shoulders. We first had them put on the sleep mask until it felt comfortable. We then secured a bicycle helmet to their head. We next had them look straight ahead and orient their head at what they felt 0° was. We guided their head with their chin until the digital magnetic angle locator showed 0°. In three conditions (Experiments 3a, 3c, and 3d) we oriented their gaze and they verbally estimated their gaze orientation. In one of the two upward gaze conditions (Experiment 3b), participants were verbally given an orientation at which to orient their gaze and they did so. After the participant moved their gaze to the requested orientation or the researcher moved their gaze to a given orientation and the participant gave their estimate, participants closed their eyes prior to moving their gaze to the subsequent orientation. They did this until they estimated all three orientations in that condition. We had them estimate a subsequent orientation from the position of the previous orientation instead of having them start from the same orientation to avoid anchoring biases (Shaffer et al., 2014, 2015). Participants were told that if their head was level and they were looking straight ahead that would be considered an orientation of 0° and if the head was oriented so they were looking directly above them on the ceiling in line with their torso that would be considered an orientation of 90°. We did not proceed until all participants clearly understood this.

### Results

Prior to analyzing the data, we removed any verbal estimates (for Experiments 3a, 3c, and 3d) where the angle of 15° was estimated at or greater than 60° or where the angle of 33° was estimated to be 90°. This is because much of the downward gaze orientation research has shown gains of 1.5 and some of the work on perceiving slanted surfaces shows that climbers cannot distinguish between hills of ~60° to 90°. This means that someone who gave a verbal estimate of greater than 60° in any direction when their head was at 15° or 90° when their head was at 33° perceived their head as tilted maximally. Also given that all participants were instructed as to what 0° and 90° is and the restrictions of the head and neck (especially in upward and downward directions), it would seem that estimates of 15° that equaled or exceeded 60° or estimates of 33° that equaled 90° are not as much overestimates of head orientation as they are a misunderstanding of what participants were asked to do. For these cases, we simply removed that estimate but kept the remaining estimates for the angles for that participant.

#### Comparing gains across head orientation tasks

Prior to analyzing each task, we first performed a Bayesian repeated-measures ANOVA comparing gains across the four different tasks using *JASP *(Version 0.19.2). The ANOVA showed that average gains were significantly different across tasks, *BF*_*10*_ = 1.41 × 10^9^ (decisive evidence for an effect), *F*(3, 87) = 21.52, *p* <.001, η^2^ = 0.48, Noninformative prior =.5. Post hoc tests indicated that upward, downward, and azimuth head orientation gains were statistically equivalent (*M*_*Azimuth*_ = 1.36 *M*_*Upward*_ = 1.558 *M*_*Downward*_ = 1.539), *BF*^*s*^_*10*_ = 0.216-.453, while the average gain when being asked to reproduce a verbally given upward head orientation (~0.619) was significantly less than azimuth, upward, and downward head orientation estimates, *BF*_*10Azimuth-VerballyGiven*_ = 942472.89 (decisive evidence), *BF*_*10Upward-VerballyGiven*_ = 33603.87 (decisive evidence), and *BF*_*10Downward-VerballyGiven*_ = 46571.43, all priors =.414. Credible Intervals for each condition are given in each of the subsequent analyses.

#### Experiment 3a: Verbal estimates of upward head orientations (15°, 33°, 51°)

Two estimates of 15° equaled or exceeded 60° (75°, 90°) and one estimate of 33° was equal to 90° so these three estimates were removed from the analyses. Each of these estimates were given by three different participants so for these three participants, we averaged their verbal estimates of the other two angles only.

**Fig. 8:**
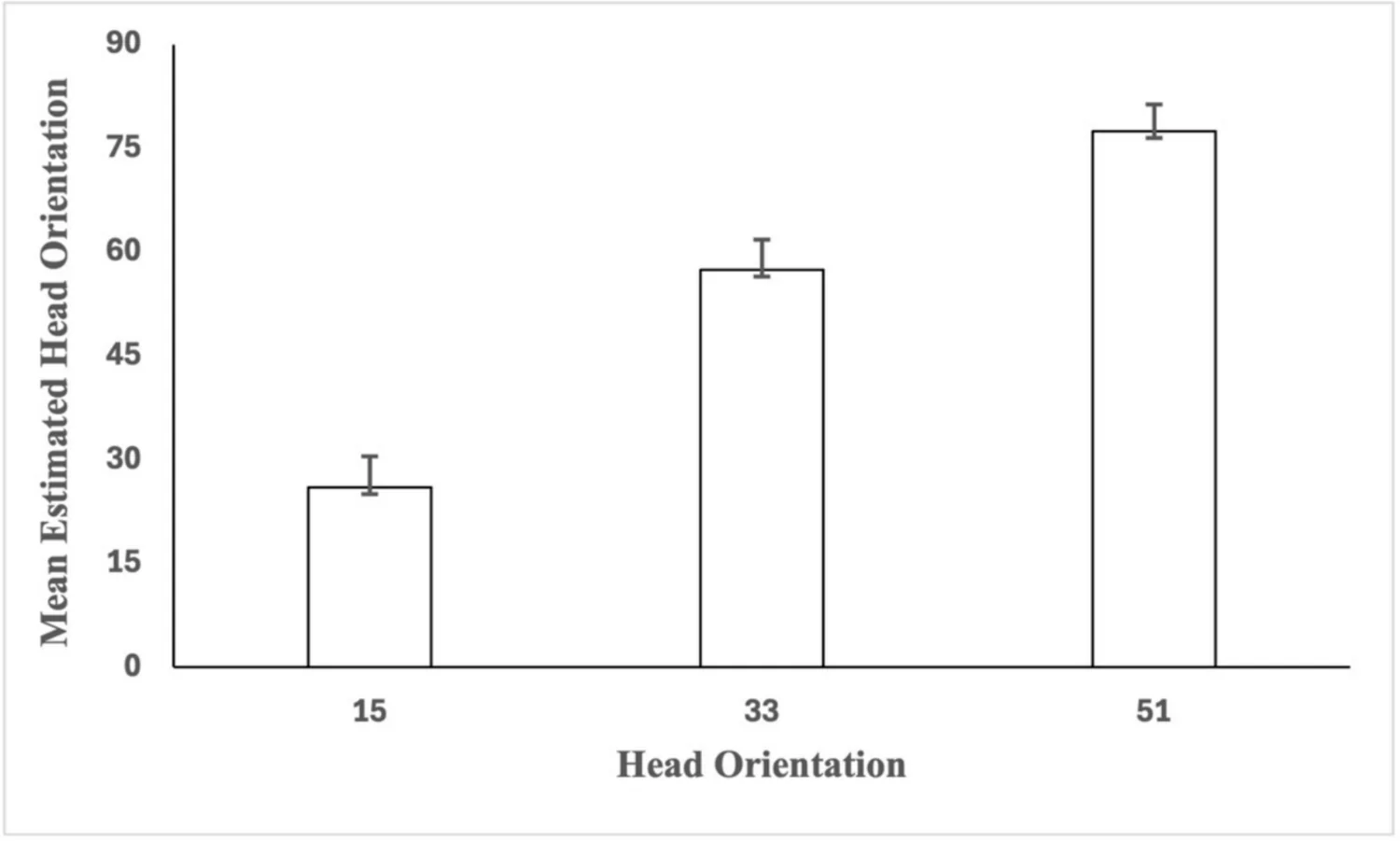
Actual head orientation versus estimated head orientation is shown. Error bars are also shown (standard errors)

Means and standard deviations for each orientation are shown in Table 7 and also shown graphically in Fig. 8. We converted observers’ verbal estimates of where their head was oriented versus where it was actually oriented to gains across the three angles they were asked to estimate (verbal estimate of head orientation/actual head orientation). To test whether observers overestimate their head orientation, we compared the values of their gains to a value of 1, which is what one would expect if observers’ estimates of head orientation were exactly equal to actual head orientation. A Bayesian one-sample *t*-test analyzing whether observers significantly overestimated their head orientation across all angles found that they did: *BF*_*10*_ = 1975.05 (decisive evidence), > Test value (one-tailed), *t*(23) = 5.27, *p* <.001, η^2^ = 0.547, *M* = 1.558 (*SD*=0.37), 95% Credible Interval: {1.339, 1.777}, Cauchy Prior with a scale of.707. This may be interpreted that it is more than 1,900 times as likely that there is a difference between verbal estimates of upward head orientation and the actual orientation than that there is no difference. We then tested the gains to a value of 1.5, the factor by which observers overestimate the slope of virtual, man-made, and geographical slopes as well as the factor by which observers overestimate downward head orientation. A Bayesian one-sample *t*-test found that there was no statistical difference between observers’ upward head orientation gains and that of downward head orientation and verbal estimates of orientations of surfaces, *BF*_*01*_ = 2.91 – anecdotal-substantial evidence in favor of the null, *t(*23) = 0.55, *p* =.294, Cauchy Prior with a scale of.707. This may be interpreted as that it is almost three times as likely that there is no difference between verbal estimates of upward head orientation and a value of 1.5 than that there is a difference.

**Table 7:** Means and standard deviations for head orientation estimates of each of the orientation positions in Experiment 3a

Angle	Mean	*SD*
15°	26°	22.11°
33°	57.42°	21.51°
51°	77.46°	18.79°

#### Experiment 3b: Upward head orienting to verbally given angles (22°, 45°, 67°)

**Fig. 9:**
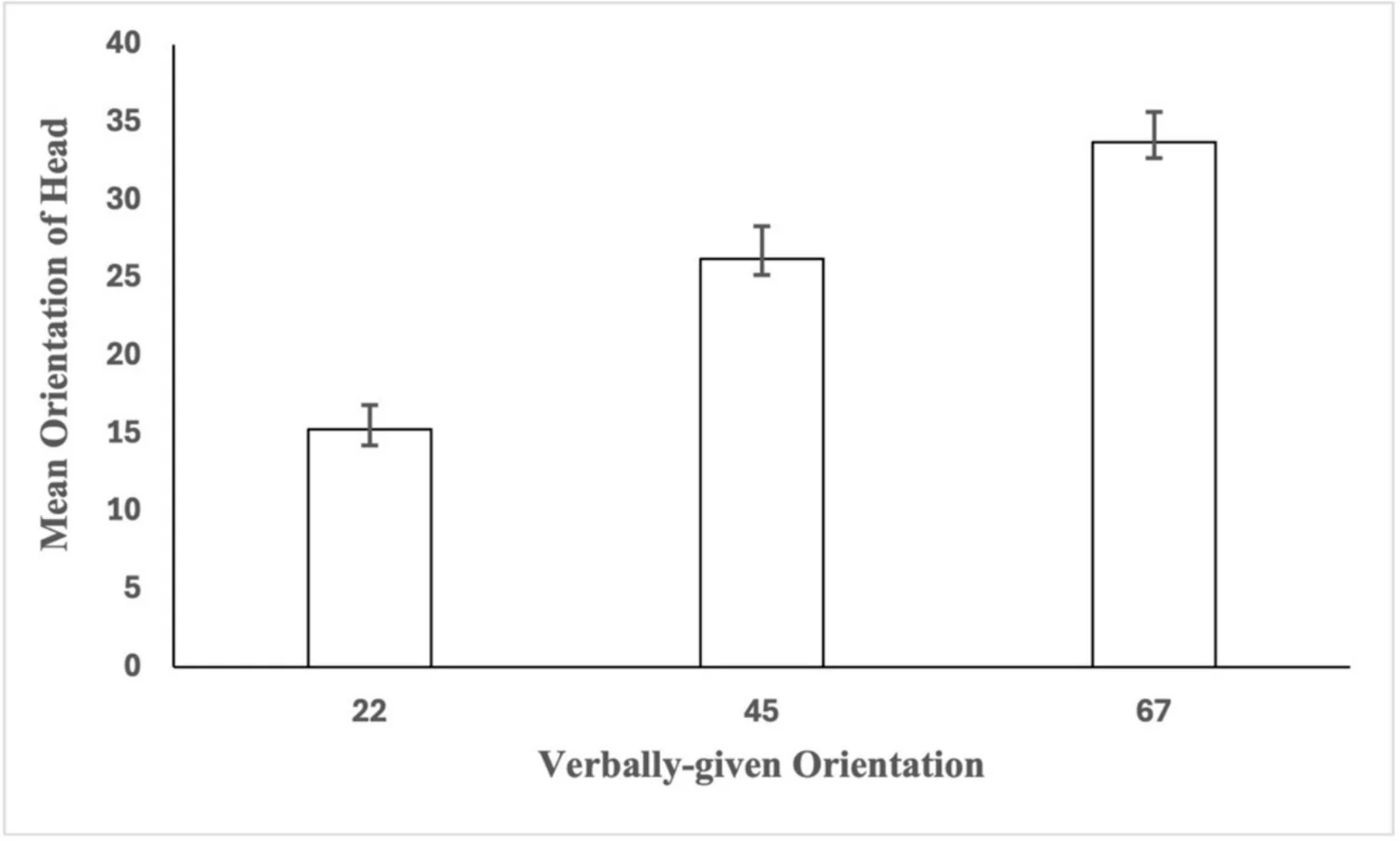
Verbally given head orientation versus where participants oriented their head orientation is shown. Error bars are also shown (standard errors)

No estimates of 22° exceeded 60° so no cases were removed. Means and standard deviations for each orientation are shown in Table 8 and Fig. 9. We converted observers’ positioning of their own head orientation to the verbally given orientation (head orientation/verbally given orientation) across the three verbally given angles. An overestimate of head orientation by a factor of 1.5 in upward head orientation would mean that if someone were asked to estimate their head orientation when we positioned their head at 30°, they would estimate it to be 45° (or their verbal estimate would be 1.5 times as great as the head orientation we asked them to estimate). Drawing from Experiment 3a, the mean gain across head orientations was 1.558, which means that if we oriented their head at 30°, they would estimate it to be ~46.74°. This also means that for what we asked of participants in the current experiment – asking them to orient their head at a verbally given angle – we should expect the inverse result. If participants overestimate upward head orientation by a factor of 1.558 as they did in Experiment 3a, when we ask them to orient their head at 30°, 30° should feel like 46.74° and their gain of where they orient their head should be 30°/46.74° =.64, much smaller than a value of 1. In order to test whether observers overestimate their head orientation, we compared the values of their gains to a value of 1, which is what one would expect if observers’ head orientation reproduction of verbally given angles were exactly equal to the verbally given angles. A Bayesian one-sample *t*-test analyzing whether observers significantly overestimated their upward head orientation (i.e., stopped their upward head orientation shallow of the verbally given orientation) across all verbally given angles found that they did: *BF*_*10*_ = 1.558 × 10^6^, < Test value (one-tailed), *t*(23) = −8.44, *p* <.001, η^2^ = 0.76, *M* =.619 and *SD* =.221, 95% Credible Interval: {0.526, 0.713}, Cauchy Prior with a scale of.707. We then tested the gains to a value of.64, the value we would predict given the results of Experiment 1a. A Bayesian one-sample t-test comparing verbal estimate of head orientation to a value of 0.64 also found that there was no statistical difference between verbal estimates and the value of 0.64, *BF*_*01*_ = 3.16 (substantial evidence for no difference), *t(*23) = −0.46, *p* =.324, Cauchy Prior with a scale of.707. Interpretation of the Bayes factor indicates that it is more than 3 times as likely that there is no difference in reproduced orientations of head orientation and a value of 0.64.

**Table 8:** Means and standard deviations for head orientation estimates of each of the requested orientations in Experiment 3b

Angle	Mean	*SD*
22°	16.23°	6.34°
45°	27.04°	11.72°
67°	34.81°	12.33°

#### Experiment 3c: Verbal estimates of downward head orientations (15°, 33°, 51°)

**Fig. 10:**
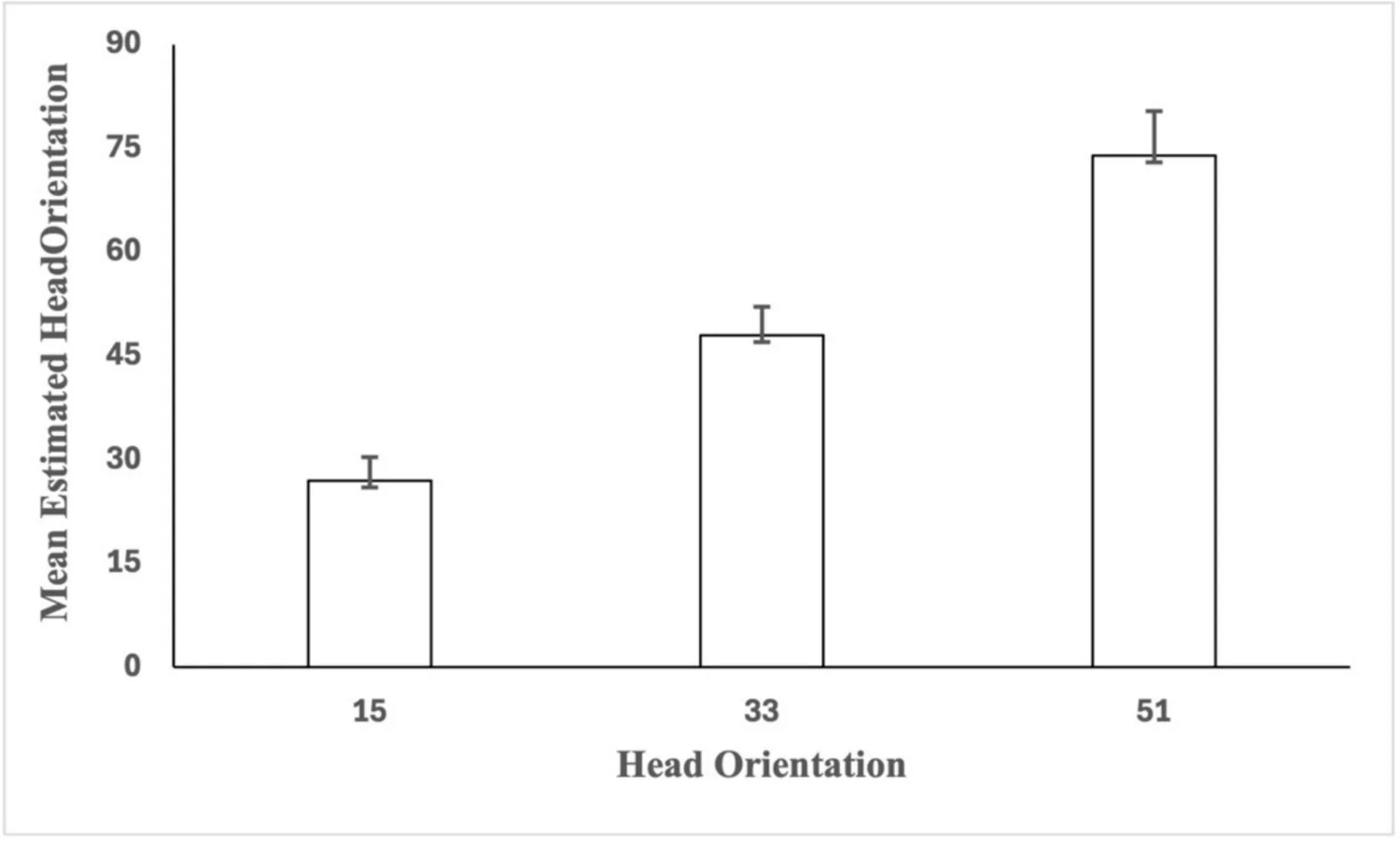
Actual head orientation versus estimated head orientation is shown. Error bars are also shown (standard errors)

One estimates of 15° equaled 60°. This estimate was removed. For that participant we averaged their verbal estimates of 33° and 51° only. Means and standard deviations are shown in Table 9 and Fig. 10. We converted observers’ verbal estimates of where their head was oriented versus where it was actually oriented to gains across the three angles they were asked to estimate (verbal estimate of head orientation/actual head orientation). To test whether observers overestimate their downward head orientation, we compared the values of their gains to a value of 1, which is what one would expect if observers’ estimates of downward head orientation were exactly equal to actual head orientation. A Bayesian one-sample *t*-test analyzing whether observers significantly overestimated their downward head orientation across all angles found that they did: *BF*_*10*_ = 539.72, > Test value (one-tailed), *t*(23) = 4.69, *p* <.001, η^2^ = 0.415, *M* = 1.539, *SD* = 0.56, 95% Credible Interval: {1.301, 1.776}, Cauchy Prior with a scale of.707. We then tested whether there was a difference between downward head orientation overestimates and upward head orientation overestimates in Experiment 3a. A Bayesian paired-samples *t*-test comparing verbal estimates of downward head orientation to the value of 1.558, the gain found in Experiment 3a for upward head orientation, also found that there was no statistical difference between verbal estimates and the value of 1.558, Bayes factor = 4.6 in favor of no difference *t*(22) = 0.17, *p* =.868, Cauchy Prior with a scale of.707.

**Table 9:** Means and standard deviations for head orientation estimates of each of the head positions in Experiment 3c

Angle	Mean	*SD*
15°	27°	16.51°
33°	47.96°	20.43°
51°	73.92°	31.56°

#### Experiment 3d: Verbal estimates of head orientation in the azimuth direction (15°, 33°, 51°)

**Fig. 11:**
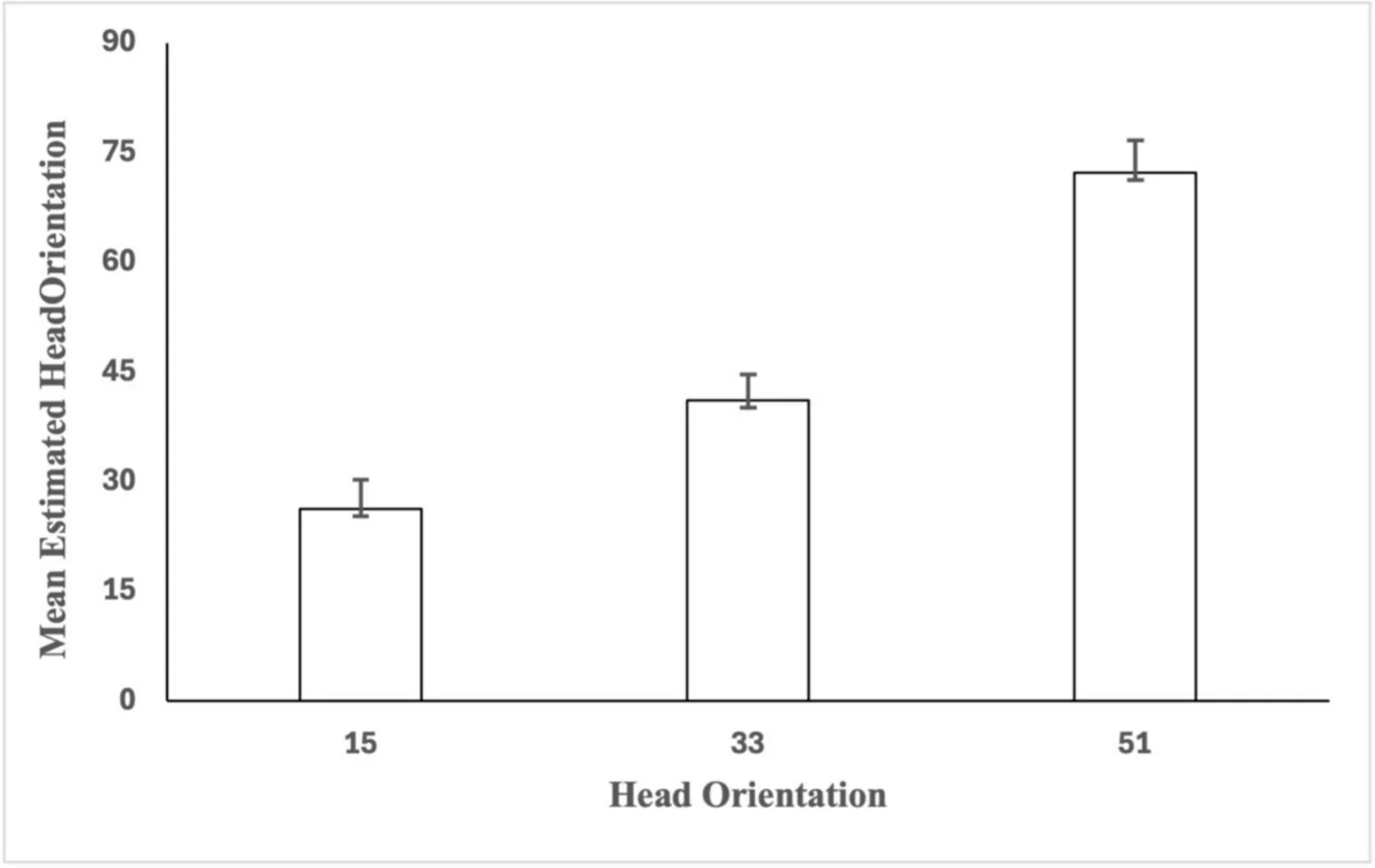
Actual head orientation versus estimated head orientation is shown. Error bars are also shown (standard errors)

Two estimates of 15° equaled or exceeded 60° (60°, 90°). These estimates were removed. For those two participants we averaged their verbal estimates of 33° and 51° only. Means and standard deviations for each orientation are shown in Table 10 and Fig. 11. We converted verbal estimates of where their head was oriented versus where it was actually oriented to gains across the three angles they were asked to estimate (verbal estimate of head orientation/actual head orientation). We first tested whether there were differences in azimuth estimates for people turning left versus those turning right. A Bayesian independent-samples t-test showed no statistical difference in azimuth direction tested, *BF*_*01*_ = 1.8, *t*(22) = 1.04, *p* =.309, *M*_*Left*_ = 1.434, *SD*_*Left*_ =.39; *M*_*Right*_ = 1.255, *SD*_*Right*_ =.45. To test whether observers overestimate their azimuth head orientation, we compared the values of their gains to a value of 1, which is what one would expect if observers’ estimates of head orientation were exactly equal to actual head orientation. A one-sample *t*-test analyzing whether observers significantly overestimated their azimuth head orientation across all angles found that they did: *BF*_*10*_ = 191.48, *t*(23) = 4.23, *p* <.001, *η*^*2*^ = 0.43, *M* = 1.36 (*SD*=0.42), 95% Credible Interval: {1.184, 1.536}, Cauchy Prior with a scale of.707.

**Table 10:** Means and standard deviations for head orientation estimates of each of the head positions in Experiment 3d

Angle	Mean	*SD*
15°	26.31°	19.13°
33°	41.1°	17.39°
51°	72.22°	4.46°

#### Experiment 3e: Head orientations to 90° upward, 90° downward, and 0° (straight ahead)

**Fig. 12:**
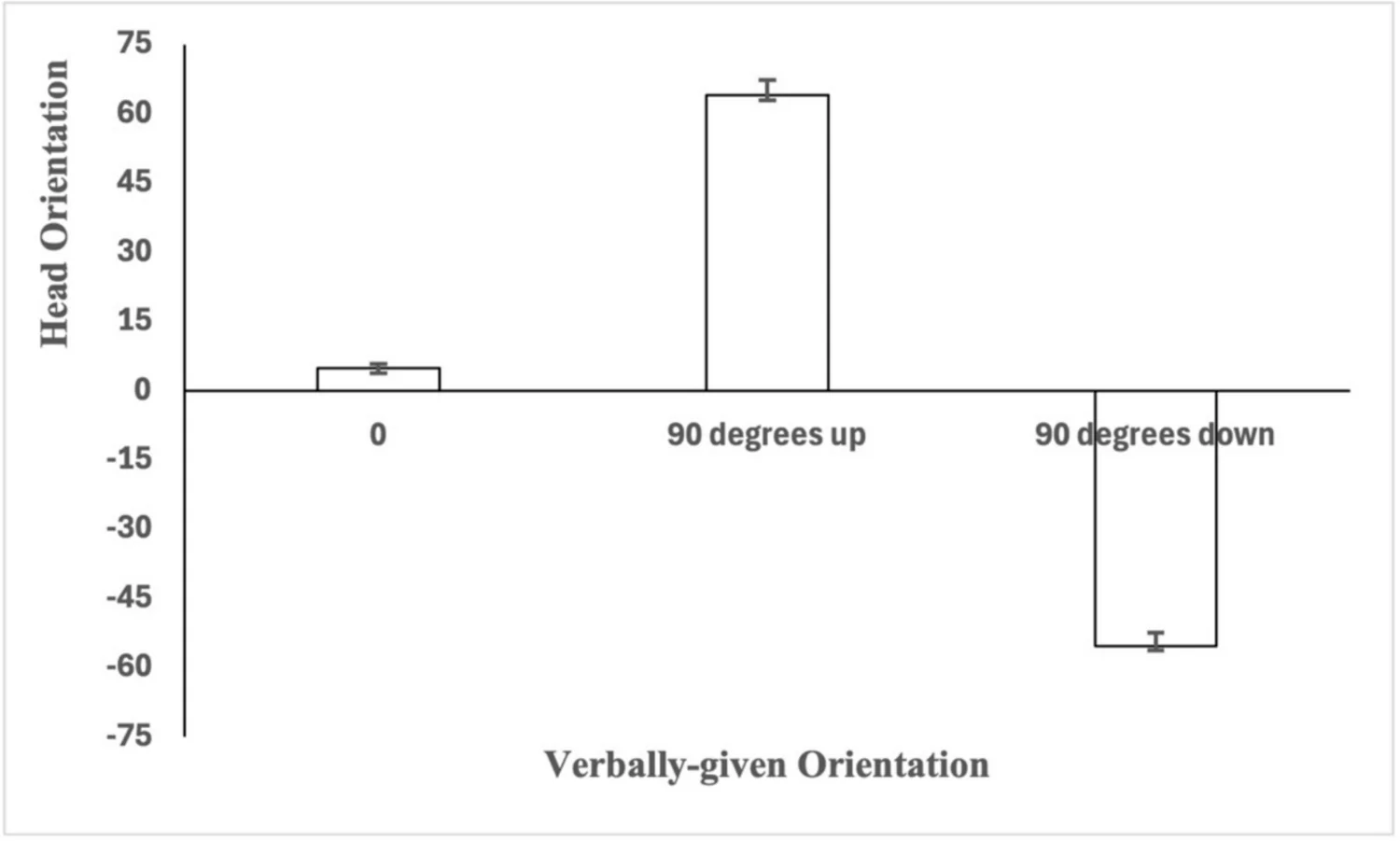
Verbally given head orientation versus where participants oriented their head orientation is shown. Error bars are also shown (standard errors)

No restrictions were placed on the data, so all 24 participants are included in the analyses. Means and standard deviations are shown in Table 11 and Fig. 12. When asked to orient their head orientation at 90° upward, participants set their head orientation at 61.93° A Bayesian one-sample *t*-test analyzing whether observers set their head orientation to significantly less than 90° found that they did, *BF*_*10*_ = 543,967.83, < Test value (one-tailed), *t*(23) = −7.9, *p* <.001, *η*^*2*^ = 0.73, 95% Credible Interval: {54.92°, 69.48°}, Cauchy Prior with a scale of.707. When asked to orient their head at 90° downward, participants oriented their head at 55.26°. A Bayesian one-sample *t*-test analyzing whether observers oriented their head to significantly less than 90° found that they did, *BF*_*10*_ = 5.52 x 10^8^, < Test value (one-tailed), *t*(23) = −11.76, *p* <.001, *η*^*2*^ = 0.857, 95% Credible Interval: {49.42°, 61.56°}, Cauchy Prior with a scale of.707. A Bayesian one-sample *t*-test analyzing whether observers oriented their head to significantly more than 0° found that the average orientation at which they oriented their head was 4.82°, *BF*_*10*_ = 1127.9, > Test value (one-tailed), *t*(23) = 5.02, *p* <.001, *η*^*2*^ = 0.53, 95% Credible Interval: {2.93°, 7.04°}, *Cauchy Prior* with a scale of.707.

**Table 11:** Means and standard deviations for head orientation estimates of each of the orientation positions in Experiment 2b

Angle	Mean	*SD*
90° Up	61.93°	16.92°
0° (Straight Ahead)	4.82°	4.83°
90° Down	55.26°	14.12°

### Discussion

In Experiment 3 we showed that the absence of vision did not seem to influence head orientation very much. While the orientations used between Experiments 1 and 3 were different, the gains seemed to remain fairly constant:: *M*_*Exp1Up*_ = 1.56, *M*_*Exp3Up*_ = 1.56, *M*_*Exp1Down*_ = 1.69, *M*_*Exp3Down*_ = 1.54, *M*_*Exp1Az*_ = 1.254, *M*_*Exp3Az*_ = 1.36, *M*_*Exp1Repro*_ =.5973, *M*_*Exp3Repro*_ = 0.619.

This is similar to the results of Shaffer et al. (2016b), who found no differences in eyes open versus eyes closed condition when participants were estimating their own body tilt. It seems likely that vestibulo-ocular mechanisms including vision, gaze, and the otolith organs all work to contribute toward a convergence of our perception gaze and head orientation. There was a bit more variability in responses in Experiment 3 than there was in Experiment 1 as is shown in Table 12. The coefficient of variations are greater for upward, downward, and azimuth orientations when participants were blindfolded compared to when participants were not. While this is most likely due to being blindfolded, we recognize that it also may reflect a lack of as much specificity of identifying orientations through otolith organs alone (Curthoys, 2020).

**Table 12:** Shown are coefficient of variations in Experiments 1 and 3 for upward, downward, and azimuth orientations

	Upward	Azimuth	Downward
Experiment 1	.239	.272	.161
Experiment 3	.333	.307	.366

#### Query concerning height of a familiar object

Just as overestimation of downward gaze orientation has interesting implications for perception of slanted surfaces and depth distances along the ground – namely predicting overestimation of slanted surfaces and underestimation of depth distance (Durgin, & Li, 2011; Li & Durgin, 2009, 2010; Li et al., 2011, 2016, Experiment 1), so too do the results found in Experiments 1a, 1b, 2a, 3a, and 3b have interesting implications for perceived upward head orientation and perceived distances above eye level. Just as previously reported compression of depth distances results from overestimating downward gaze declination, one should expect a perceived expansion of elevation distances from overestimating upward head orientation with eyes straight forward by a factor in the range of ~1.56 (Experiment 1a/3a) to 1.7 (found in Experiment 2a).

Indirect evidence in support of this idea comes from the pole-matching tasks of Li et al. (2011), whose participants matched the height of four poles that varied in height from 3.75 to 22.5 m by adjusting their forward/backward distance to the pole. The matching estimates fit well to a linear model with a gain of 1.5 (depth distance away from the pole: vertical distance). They performed a similar task with a lamp post and found a gain of 1.47. Finally, they also performed an angular direction task asking their participants to adjust their position until they felt like their gaze was oriented at 45° when looking at a 35-m tall column holding a water tank. Participants stopped when their gaze was oriented at 30.7°, suggesting a gaze inclination expansion by a factor of ~1.5. The pole-matching gain of 1.5 fits the gains of 1.56 and 1.7 found in Experiments 1a/3a and 2a, shown statistically in both *Results* sections for each Experiment. The lamp post-matching gain of 1.47 is also statistically similar to gains of 1.56 and 1.7 respectively, *BF*_*01*_ = 0.33, *t(*29) = 1.45, *p* =.158 and *BF*_*01*_ = 0.21, *t(*24) = 1.62, *p* =.118. Similar work has found similar matching gains of depth distance on the ground to height ranging from ~1.5 to ~1.7 (Higashiyama & Ueyama, 1988; Kammann, 1967).

#### Survey questionnaire

While we have shown that the factor by which observers overestimate upward head orientation is similar to previous work showing similar factors by which observers match depth or horizontal distances on the ground to height, we wanted to directly test whether our findings from Experiments 1a, 2a, and 3a could be connected to verbal estimates of the height of a familiar object. Upward head orientation overestimation predicts an expansion of perceived distance when looking upward. Therefore we tested whether verbally estimated distances to a familiar suspended object are overestimated consistent with perceived upward head orientation found in the current work.

We conducted a survey asking participants questions concerning general orientations, gaze orientations, and one question concerning their memory of the height of a familiar object – this was our target question. The other questions acted as fillers. We then tested whether head orientation might be tied to memory for the height of that object.

### Method

#### Participants

The same 33 participants who participated in Experiment 2 answered several other questions concerning heights and head orientation angles.

#### Procedure

The question of focus was asking participants to estimate the height from the ground of the center of the middle lens of a traffic light. The traffic light housing is 5.18 m from the ground. The middle lens is 5.64 m from the ground.

### Results

We removed two estimates that were outside five standard deviations of the mean (91.44m and 45.72m). We then performed a Bayesian one-sample *t*-test comparing participants’ verbal estimate of the height of a traffic light to 5.64 m and found that they significantly overestimated the height of the traffic light, *BF*_*10*_ = 20.88, *t*(30) = 3.15, *p* =.002, *η*^*2*^ =.07, *M* = 10.73, *SD* = 8.99, 95% Credible Interval: {7.43, 14.02}, Cauchy Prior with a scale of.707. We then compared the actual height multiplied by the factor by which observers overestimated upward head orientation in both Experiments 1a/3a (1.56) and in Experiment 2a (1.7). After multiplying 1.56 and 1.7 times the actual height of the traffic light, these numbers are 8.8 m and 9.588 m, respectively. Two Bayesian one-sample *t*-tests showed that the overestimation of the height of the traffic light matched the factor by which participants overestimated their upward head orientation applied to the traffic light height (1.56 – Experiments 1a/3a), *BF*_*01*_ = 2.74, *t*(30) = 1.19, *p* =.243, and (1.7 – Experiment 2a, *BF*_*01*_ = 4.15, *t*(30) = 0.704, *p* =.487, Cauchy Prior =.707 for both analyses.

The survey questionnaire showed that perceived upward head orientation seems to be directly tied to verbal estimates of the heights of familiar suspended objects from memory. While we did not directly manipulate head orientation, height, and size of suspended objects in the current work, previous work has shown that suspended objects are perceived as smaller than equally sized versions of those objects at eye level (Granrud et al., 2003). For instance, traffic light lens diameters are 0.31m. However, from memory and when looking directly at them people underestimate their size by ~30% (Granrud, et al., 2003; McBeath et al., 1993). Similarly, one can fit ~1.9 basketballs side-by-side in a basketball hoop but people estimate that an average of 1.5 basketballs can fit side-by-side into the hoop (McBeath et al., 1993). If one is unfamiliar with the actual size of an object even if we are familiar with the object and have seen it many times before, size distance scaling may not apply and we may have to rely on its *assumed size* based on estimates of its size and distance from us (or height in the case of suspended objects) (Granrud et al., 2003; McBeath et al., 1993; Shaffer et al., 2008a, b).

In some of our previous work, among a set of data in a questionnaire, we asked twenty-six participants from the Ohio State University at Mansfield who participated in fulfillment of an Introductory Psychology requirement to estimate from memory the width and height of a speed limit sign on a freeway in Ohio with which they were familiar. Each participant was required to sign a consent form for the study. Informed consent was obtained for all participants. This study was approved by The Ohio State University Behavioral and Social Sciences Institutional Review Board (Study Number: 2023B0282). The 26 participants were independent of any of those in the current work’s previous studies. Bayesian one-sample *t*-tests showed that they significantly underestimated both the width and height of a speed limit sign, Width = 1.22 m (Ohio Department of Transportation (ODT), 2005), *BF*_*10*_ = 5.26 × 10^15^, < Test value (one-tailed), *t*(25) = −23.43, *p* <.001, *η*^*2*^* =.84*, *M* = 0.42 m, *SD* = 0.18 m, 95% Credible Interval: {0.35, 0.49}, Cauchy Prior with a scale of.707; *Height* = 1.524 m (ODT, 2005), *BF*_*10*_ = 100,773.64, < Test value (one-tailed), *t*(25) = −6.88, *p* <.001, *η*^*2*^ =.31, *M* = 0.795 m, *SD* = 054 m, 95% Credible Interval: {0.58, 1.01}, Cauchy Prior with a scale of.707. While the relationship between assumed size from memory, height, distance, and physical size is a more complicated picture than we can directly test here, the assumed size of suspended objects where upward head orientation is required like traffic light lenses, basketball hoops, and speed limit signs on freeways seems like it may be determined at least in part by the overestimation of height, which may be influenced by our overestimation of upward head orientation in addition to other factors.

## Summary

We have investigated perceived head orientation in upward and downward (elevation) and azimuth coordinates with the same individuals. We have shown that upward head orientation is overestimated by a factor of between 1.5 and 1.7 – the same factor by which downward head orientation was overestimated in the current study (1.69 – Experiment 1c) and is statistically similar to previous work on downward gaze overestimates (~1.5, Durgin & Li, 2011; Li & Durgin, 2009). This work converges with gaze orientation work showing overestimates of both elevation (up and down) and azimuth (left and right) orientations in the same observers. These systematic overestimates seem to be a product of both visual and nonvisual head orientation mechanisms. Our work shows that upward head orientation is perceived to be farther “up” than actual. This, in turn, has predictable implications for perceived distances when looking upward, which fit our and other’s findings of perceived expansion of upward distances (height) quite well. These findings coupled with work showing that suspended objects are perceived as smaller than their eye-level equivalents suggest that upward head orientation overestimates may play a role. We have also shown the “perceptual boundaries” for upward and downward head orientation nicely fit the scale expansion factors by which head orientation is overestimated. This also gives us a perceived spatial map within which our gaze and gaze/head orientation regularly operate. There are many occasions where one looks down prior to looking up or has to do both to evaluate the height of objects starting on the ground (and below eye level). Therefore, a common scale expansion for elevation more generally appears to be a useful perceptual regularity for examining surfaces, distances along the ground, and heights that operates irrespective of gaze/head orientation in the elevation direction and has reliable predictions for how we perceive the world.

## Data Availability

Data for Experiments 1 (a–d), Experiments 2 (a and b), Experiments 3 (a–e), and the survey questionnaire may be accessed at: https://osf.io/m5gpy/ None of the experiments was preregistered.

## Footnotes

1 We used G*Power 3.1.9.6 (Faul et al., 2007) rather than a software for more sophisticated repeated analyses like GLIMMPSE (Guo et al., 2013) for determining sample size as we were not conducting a longitudinal study, and our reasoning behind using a repeated-measures design is more about the methodology than the statistical design. Additionally, we did not use covariates or mixed models, and had only a one-way repeated-measures ANOVA with post hoc tests planned. The remainder of our analyses were not repeated measures.

2 Partial $${\eta }^{2}\frac{\mathrm{F}}{\mathrm{F}+ {\mathrm{df}}_{\mathrm{W}}}= .19315$$; Cohen’s *f* = $$f=\sqrt{{\mathrm{np}}^{2}}=.4395$$

3 Though we used* f* and *d* values for inputs for our G*Power analyses, we used ${\eta }^{2}$ values for all of our results, consistent with the recommendations of Correll et al. (2020) and Dienes (2024). $\text{For translations}:{ \eta }^{2}= \frac{{f}^{2}}{1+ {f}^{2}}$, and ${\eta }^{2}= \frac{{d}^{2}}{4+ {d}^{2}}$

4 Bayes factors were calculated as evidence for both the null (*BF*_*01*_ = P(H_0_)/P(H_A_)) and alternative (*BF*_*10*_ = P(H_A_)/P(H_0_) hypotheses. According to Wetzels et al. (2011), a Bayes factor of 0–1 is no evidence for either the null (H_0_) or the alternative hypothesis (H_A_), 1–3 is anecdotal evidence for the tested hypothesis (probability of the hypothesis in the numerator of the equations above), 3–10 is substantial evidence for the tested hypothesis, 10–30 is strong evidence for the tested hypothesis, 30–100 is very strong evidence for the tested hypothesis, and 100 is decisive evidence for the tested hypothesis. However, it really depends on the probability of which hypothesis one places in the numerator/denominator.

5 We reported credible intervals rather than confidence intervals consistent with misinformation confidence intervals provide and the recommendations of Morey et al. (2016).
